# Prevalence and risk factors of osteosarcopenia: a systematic review and meta-analysis

**DOI:** 10.1186/s12877-023-04085-9

**Published:** 2023-06-15

**Authors:** Tianjin Huang, Chen Li, Faxiu Chen, Dunan Xie, Chuhua Yang, Yuting Chen, Jintao Wang, Jiming Li, Fei Zheng

**Affiliations:** 1 Medical College of Nanchang University, Nanchang, China; 2 Jiangxi Provincial People’s Hospital, The First Affiliated Hospital of Nanchang Medical College, Nanchang, China

**Keywords:** Osteosarcopenia, Sarcopenia, Prevalence, Systematic review, Meta-analysis

## Abstract

**Background:**

Osteosarcopenia is a syndrome with a concomitant presence of both sarcopenia and osteopenia/osteoporosis. It increases the risk of frailty, falls, fractures, hospitalization, and death. Not only does it burden the lives of older adults, but it also increases the economic burden on health systems around the world. This study aimed to review the prevalence and risk factors of osteosarcopenia to generate important references for clinical work in this area.

**Methods:**

Pubmed, Embase, Cochrane Library, Web of Science, CNKI, Wanfang, CBM, and VIP databases were searched from inception until April 24th, 2022. The quality of studies included in the review was evaluated using the NOS and AHRQ Scale. Pooled effects of the prevalence and associated factors were calculated using random or fixed effects models. *Egger*’s test, *Begg*’s test, and funnel plots were used to test the publication bias. Sensitivity analysis and subgroup analysis were conducted to identify the sources of heterogeneity. Statistical analysis was performed using Stata 14.0 and Review Manager 5.4.

**Results:**

A total of 31 studies involving 15,062 patients were included in this meta-analysis. The prevalence of osteosarcopenia ranged from 1.5 to 65.7%, with an overall prevalence of 21% (95% *CI*: 0.16–0.26). The risk factors for osteosarcopenia were female (*OR* 5.10, 95% *CI*: 2.37–10.98), older age (*OR* 1.12, 95% *CI*: 1.03–1.21), and fracture (*OR* 2.92, 95% *CI*: 1.62–5.25).

**Conclusion:**

The prevalence of osteosarcopenia was high. Females, advanced age, and history of fracture were independently associated with osteosarcopenia. It is necessary to adopt integrated multidisciplinary management.

**Supplementary Information:**

The online version contains supplementary material available at 10.1186/s12877-023-04085-9.

## Background

The concept of osteosarcopenia (OS) was first proposed by Duque and colleagues [1] as the presence of low muscle mass and function (sarcopenia) together with low bone mineral density (BMD), i.e. osteopenia or osteoporosis [2–5]. Osteoporosis is a systemic skeletal disease characterized by loss of bone mass and microstructural integrity, both of which are closely associated with osteoporotic fractures. Based on the international consensus on osteopenia/osteoporosis by the World Health Organization (WHO), osteoporosis can be defined as having a T-score ≤ -2.5 standard deviation (SD) lower than the mean BMD of the same-sex reference population. Osteopenia can also be diagnosed in an individual with a T-score between − 1.0 and − 2.5 [6]. Sarcopenia is a skeletal muscle disease characterized by a decline in muscle mass, accompanied by muscle strength and/or physical function [7, 8]. Sarcopenia was first proposed by Professor Rosenberg in 1989 [9]. In 2010, the European Working Group on Sarcopenia in Older People (EWGSOP) published the first consensus on the definition. The diagnosis of sarcopenia can be made based on the presence of low muscle mass and low muscle function (strength or performance) [10]. Following that, the International Working Group for Sarcopenia (IWGS), the Asian Working Group for Sarcopenia (AWGS) and the US Foundation for the National Institutes of Health (FNIH) also announced a consensus [11, 12]. In 2018, EWGSOP updated the consensus by suggesting that muscle mass should also be included in the definition of sarcopenia [13]. Subsequently, in 2019, AWGS also updated its expert consensus on sarcopenia, and put forward the concept of “possible sarcopenia” to make early lifestyle intervention possible [14].

In recent years, increasing evidence suggests a close connection between muscles and bones. Apart from mechanical interactions, they are also affected by endocrine factors and have extensive genetic and molecular associations [15]. The “mechanostat” theory states that muscles exert mechanical force on bones, and when these forces exceed a certain threshold, bone turnover shifts from resorption to formation [16]. Conversely, long-term lack of exercise leads to decreased mechanical stimulation, resulting in decreased muscle mass and function, as well as reduced bone density [17]. Genome-wide association studies (GWAS) emphasize several genes that may have pleiotropic effects on bones and muscles, including the myocyte enhancer factor-2 C (MEF2C) and sterol regulatory element-binding transcription factor 1 (SREBF1) [18]. Additionally, polymorphisms in the gene family of growth/differentiation factor 8 (GDF8), glycine-N-acyltransferase(GLYAT), methyltransferase-like 21 C (METTL21C), and peroxisome proliferator-activated receptor gamma coactivator 1-alpha(PGC-1α) are also associated with loss of muscle and bone mass [1, 19]. Bones and muscle tissues can also interact with each other through autocrine, endocrine, and paracrine mechanisms [20]. Muscles secrete factors that can affect other tissues, called “myokines” which participate in bone metabolism. Some myocellular factors (such as insulin-like growth factor-1, osteocalcin, irisin, bone morphogenetic protein, follicle-stimulating hormone,and interleukin-15) have synthetic metabolic effects on the skeleton, whereas other myokines (such as myostatin and interleukin-6) have negative regulatory effects on bone function [21]. Conversely, “osteokines” (such as osteocalcin, osteoprotegerin, and sclerostin) secreted by bone cells have regulatory effects on muscle synthesis and breakdown metabolism [21]. The accumulation of muscle and bone marrow fat is one of the markers of osteoporosis and sarcopenia [22], and therefore also a marker of decreased bone and muscle mass in osteosarcopenia [1]. Studies have shown that lipid infiltration can induce local lipotoxicity, leading to cellular dysfunction, reduced bone formation, and impaired muscle synthesis and metabolism [23]. The coexistence of two musculoskeletal disorders is strongly associated with frailty, falls, fractures, hospitalization, and mortality [24, 25]. In Korea, the prevalence of osteosarcopenia in elderly patients over 60 years old with hip fractures was 27.2%. The mortality rate was 1.8 times higher than in patients without osteosarcopenia [26]. According to reports, there is a correlation between osteosacopenia and chronic diseases, including diabetes, digestive diseases, inflammatory arthritis, kidney dysfunction, depression [27], heart disease, polycystic ovary syndrome(PCOS) [28], and hyperthyroidism [29]. These comorbidities may affect the pathological andphysiological mech-anisms of osteosarcopenia, and increase the risk of musculoskeletal health damage.

In the past few years, the increased interest in the field of osteosarcopenia among clinicians and researchers has led to the publication of numerous studies on the prevalence and risk factors of osteosarcopenia. To date, there are two systematic reviews on the epidemiology of osteosarcopenia. Yoo et al. summarized the epidemiology of osteosarcopenia in Korea [26]. Nielsen et al. reported a prevalence of osteosarcopenia of 5–37% depending on the classification of sarcopenia and whether participants were initially classified as having sarcopenia or osteoporosis [30]. However, there is still a lack of studies on the aggregation of global prevalence. Furthermore, no studies have investigated the factors contributing to the heterogeneity of the prevalence estimates of osteosarcopenia through a meta-analysis. Therefore, this systematic review aimed to generate comprehensive findings on the prevalence and risk factors of osteosarcopenia based on evidence from epidemiological cross-sectional surveys in order to provide a theoretical basis for the prevention and treatment of osteosarcopenia.

## Methods

This systematic review and meta-analysis were performed according to the Preferred Reporting Items for Systematic Review and Meta-Analysis (PRISMA) guidelines [31]. It was also registered with PROSPERO (CRD42022331412).

### Search strategy

Eight databases including Pubmed, Embase, Cochrane Library, Web of Science, China National Knowledge Infrastructure (CNKI), Wanfang, China Biomedical Literature Database (CBM), and VIP database were searched to obtain relevant literature. The references in the literature were also screened for relevant studies. The retrieval time ranged from the establishment of the database to April 24th, 2022. The English search terms were based on a combination of relevant MeSH terms, i.e. “osteoporosis”, “sarcopenia”, “risk factors”, and “prevalence”. It was also supplemented by the method of literature traceability to ensure that as much literature was searched as possible.

### Study selection

The inclusion criteria for the review included: (1) Observational study designs such as case-control, cross-sectional, and longitudinal cohort studies; (2) The study population was the general adult population; (3) Prevalence with or without risk factors of osteosarcopenia was reported in the study; (4) Clear and valid diagnosis of osteopenia/osteoporosis and sarcopenia; (5) Relevant data on the prevalence of osteosarcopenia, as well as relevant risk factors in the form of odds ratio (*OR*, 95% *CI*) were provided or could be generated from the raw data in the study.

The exclusion criteria included: (1) Reviews, article reviews, lectures, case reports, conference abstracts, and animal experiments; (2) Poor data quality, small sample data, repeated publications, or similar studies; (3) Data with obvious errors, incomplete data that cannot be utilized, poor quality literature, and inability to obtain the data needed for the study.

Two authors independently screened the titles and abstracts of all articles. The screening results of the two reviewers were compared. Any differences were discussed to obtain a consensus. Full-text reviews and data extraction were then independently performed by the same two reviewers. The results were again compared and discussed for agreement. If there are any unresolved discrepancies between the two reviewers at any stage, a third reviewer was consulted.

### Data extraction

The following data were extracted using Excel spreadsheets by two reviewers independently, i.e. authors, year of publication, title, study design, region, sample size (including male, female, sex ratio), diagnostic criteria of osteoporosis and sarcopenia, the prevalence of osteosarcopenia, as well as associated risk factors.

### Quality assessment

For the quality assessment of a cross-sectional study, the 11-item criteria recommended by the US Agency for Healthcare Quality and Research (AHRQ) was used. A score of 0 to 3 indicates low quality, 4 to 7 indicates medium quality, and 8 to 11 indicates high quality. For cohort studies, the Newcastle-Ottawa Scale (NOS) [32] was used. The quality of the study was evaluated by eight items under the three categories of participant selection, comparability of study groups, and ascertainment of outcome or exposure. A score of ≥ 7 is classified as high-quality literature.

### Statistical analysis

In this meta-analysis, the combined prevalence of osteosarcopenia and 95% *CI* were determined. The heterogeneity was tested using *I*^*2*^. The data were considered significantly heterogeneous if *I*^*2*^ > 50% and the random effects model would be used. Otherwise, the fixed-effects model was used. Sensitivity analysis and subgroup analysis were used to explore the sources of heterogeneity. The risk of publication bias was assessed by *Egger*’s and *Begg*’s tests as well as funnel plots. All the statistical analysis was conducted using STATA 14.0 (Stata Corporation, College Station, TX). However, the *OR*s and 95% *CI*s for pooled risk factors were calculated using Review Manager 5.4. A *p*-value of < 0.05 was considered statistically significant.

## Results

### Study selection

**Fig. 1:**
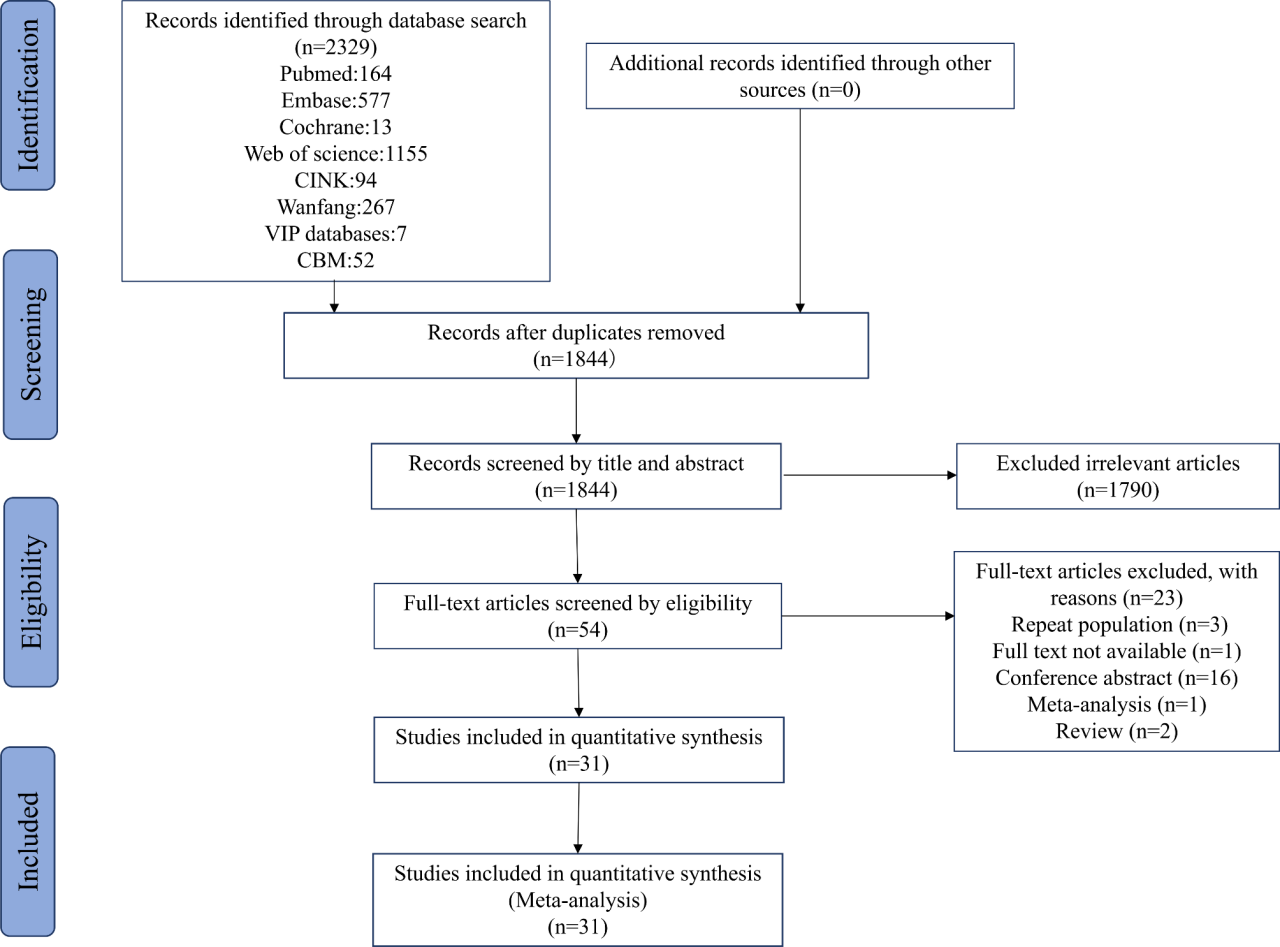
The study selection process

From the total of 2329 relevant articles retrieved, 1844 articles remained after removing duplicate literature. Following that, another 1790 studies were excluded after reviewing the titles and abstracts. The remaining 54 literatures were evaluated by reviewing the full text. Finally, only 31 studies with a total of 15,062 patients [4, 25, 33–61] were included in the final review (Fig. 1).

### Study characteristics [4, 25, 33–61]

The 31 studies were published between 2013 and 2022 with a sample size of 68 to 2353 participants. The mean age of the participants ranged from 64.1 to 84.8. The vast majority of studies were conducted in Asia (*n* = 13) [34, 35, 40–44, 48, 50–52, 54, 56] while the remaining included eight studies [25, 33, 36–38, 49, 57, 58] from Europe, six studies [4, 39, 45, 46, 55, 59] from Oceania, and four studies [47, 53, 60, 61] from the Americas. As for the study population, all 13 studies evaluated hospitalized patients (*n* = 13) [33, 37, 39–42, 47, 50–52, 57, 58, 61] and community-dwelling (*n* = 13) [4, 25, 34–36, 38, 44–46, 48, 53, 54, 60] patients while five include outpatient participants. Regarding the diagnostic criteria for sarcopenia, AWGS was used in eight studies [40, 41, 43, 44, 48, 52, 54, 56] while EWGSOP was applied in 16 studies [4, 25, 33, 35–39, 45–47, 49, 53, 55, 57, 59]. In addition, two studies [58, 61] applied the criteria by FNIH, and the other three studies [42, 50, 51] applied Japan Society of Hepatolog (JSH). The remaining two studies [34, 60] used two other sets of diagnostic criteria. In addition, some studies consider osteosarcopenia to be present in individuals with sarcopenia and low BMD (osteopenia or osteoporosis) [4, 25, 33, 35, 38, 39, 44, 45, 47, 49, 52, 53, 55, 57, 59, 61], while other scholars define osteosarcopenia as the coexistence of osteoporosis and sarcopenia [34, 36, 37, 40–43, 46, 48, 50, 51, 54, 56, 58, 60]. (Table 1).

**Table 1:** Characteristics of the studies

Author (Year)	Region	Study design	Study population	Age (yrs)	BMI (kg/m^2^)	Sample size			Prevalence *n* (%)			Diagnostic criteria of osteoporosis	Diagnostic criteria of sarcopenia	Definition of osteosarcopenia	Risk Factors (*OR*, 95% *CI*)
						Total	Male	Female	Total	Male	Female				
Pourhassan (2021) [33]	Germany	Cross- sectional	Hospitalized patients	75.1 ± 10.8	27.3 ± 5.3	572	449	123	47 (8)	-	-	WHO	EWGSOP2	Osteopenia/Ost-eoporosis + SP	-
Kobayashia (2020) [34]	Japan	Cross- sectional	Community-dwelling	71.4 ± 5.3	23.6 ± 3.5	427	205	222	36 (8.4)	9 (4)	27 (13)	Others	Others	Osteoporosis + SP	-
Fahimfar (2020) [35]	Iran	Cross- sectional	Community-dwelling	-	27.1	2353	1148	1205	708 (30.1)	339 (29.5)	369 (30.6)	WHO	EWGSOP	Osteopenia/Ost-eoporosis + SP	-
Nielsen (2020)^a^ [36]	Danish	Cross- sectional	Community-dwelling	75.0 ± 7.0	25.9	529	232	297	8 (1.5) 7 (1.4)	4 (1.2)	4 (1.3)	WHO	EWGSOP2 CCS	Osteoporosis + SP	-
Reiss (2019) [37]	Austria	Cross- sectional	Hospitalized patients	80.6 ± 5.5	26.5 ± 4.6	141	57	84	20 (14.2)	6 (10.5)	14 (16.6)	WHO	EWGSOP	Osteoporosis + SP	-
Drey (2016) [38]	Germany	Cross- sectional	Community-dwelling	-	-	68	21	47	19 (27.9)	6 (28.5)	13 (27.6)	WHO	EWGSOP	Osteopenia/Ost-eoporosis + SP	-
Huo (2015) [39]	Australia	Cross- sectional	Hospitalized patients	-	27.9 ± 6.1	680	224	455	258 (37)	45 (20.1)	213 (47.8)	WHO	EWGSOP	Osteopenia/Ost-eoporosis + SP	Female (7.48, 3.9–14.0); older age (1.08, 1.0-1.1); higher risk for depression (2.66, 1.1–6.3), maternal hip fracture (2.2; 0.9–5.5); hyperparathyroidism (2.17, 0.5-9.0)
Okamura (2020) [40]	Japan	Cross- sectional	Hospitalized patients	77.1 ± 6.7	-	276	-	276	54 (19.6)	-	54 (19.6)	JOS	AWGS	Osteoporosis + SP	BMI (1.71, 1.46–2.00)
Scott (2019) [4]	Astralia	Cohort	Community-dwelling	76.7 ± 5.4	27.9 ± 3.9	1575	1575	-	131 (8.3)	131 (8.3)	-	WHO	EWGSOP	Osteopenia/Ost-eoporosis + SP	-
Yoo (2018) [41]	Korea	Cohort	Hospitalized patients	77.8 ± 9.7	22.2 ± 3.8	324	78	246	93 (28.7)	31 (39.7)	62 (25.2)	WHO	AWGS	Osteoporosis + SP	-
Saeki (2021) [42]	Japan	Cross- sectional	Hospitalized patients	68.0	22.3	117	21	96	18 (15.4)	-	-	WHO	JSH	Osteoporosis + SP	-
Inoue (2022) [43]	Japan	Cross- sectional	Outpatient	76.5 ± 7.2	23.7 ± 4.3	495	155	340	55 (11.1)	15 (9.7)	40 (11.7)	JOS	AWGS2	Osteoporosis + SP	-
Salech (2021) [25]	Chile	Cross- sectional	Community-dwelling	72.0 ± 6.7	-	1119	351	768	183 (16.4)	52 (14.8)	131 (17.1)	WHO	EWGSOP	Osteopenia/Ost-eoporosis + SP	-
Pang (2021) [44]	Singapore	Cross- sectional	Community-dwelling	-	-	463	204	259	22 (4.7)	-	-	WHO	AWGS2	Osteopenia/Ost-eoporosis + SP	Increasing age (1.11, 1.07–1.15), male (0.2, 0.09–0.47), Chinese (0.24, 0.09–0.66), BMI (0.74, 0.65–0.84)
Hassan (2020)^a^ [45]	Australia	Cross- sectional	Community-dwelling	79.0 ± 7.5	27.3/27.1	558	195	363	130 (23) 200 (36)	32 (16.4)	98 (26.9)	WHO	EWGSOP2 EWGSOP	Osteopenia/Ost-eoporosis + SP	-
Kirk (2020) [46]	Australia	Cross- sectional	Community-dwelling	76.0	27.4	484	147	337	25 (5.1)	-	-	WHO	EWGSOP2	Osteoporosis + SP	-
Intriago (2020) [47]	Ecuadoria	Cross- sectional	Hospitalized patients	66.0 ± 10.0	26.7 ± 4.1	92	9	83	52 (56)	3 (33.3)	49 (59)	WHO	EWGSOP	Osteopenia/Ost-eoporosis + SP	-
Wang (2015) [48]	China	Cross- sectional	Community-dwelling	-	23.3 ± 3.0	316	164	152	40 (12.7)	17 (10.4)	23 (15.1)	WHO	AWGS	Osteoporosis + SP	Age (4.8, 3.05–10.76), female (2.6, 1.18–2.76), comorbidity (3.71, 1.61–10.43)
Hamad (2020) [49]	Turkey	Cross- sectional	Outpatient	64.1 ± 8.9	30.0 ± 5.1	140	-	140	90 (64.3)	-	90 (64.3)	WHO	EWGSOP	Osteopenia/Ost-eoporosis + SP	-
Saeki (2019)^a^ [50]	Japan	Cross- sectional	Hospitalized patients	70.5	23.7	142	90	52	31 (21.8)	14 (15.5)	17 (32.6)	WHO	JSH AWGS EWGSOP2	Osteoporosis + SP	-
Saeki (2020) [51]	Japan	Cross-sectional	Hospitalized patients	70.0	23.1	291	137	154	49 (16.8)	16 (11.6)	33 (21.4)	WHO	JSH	Osteoporosis + SP	BMI (0.821, 0.726–0.929), IGF-1 (0.98, 0.964–0.996), vertebral fracture (3.306, 1.439–7.596), frailty (9.837, 4.199–23.043), PTH-intact (pg/mL) (1.017, 1.005–1.030)
Lin (2021) [52]	China	Cross- sectional	Hospitalized patients	-	23.9 ± 3.8	1199	-	1199	363 (30.3)	-	363 (30.3)	WHO	AWGS2	Osteopenia/Ost-eoporosis + SP	Fracture (5.81, 0.76–44.3)
Miriam T (2021) [53]	Mexica	Cross- sectional	Community-dwelling	70.3 ± 10.8	-	825	189	636	73 (8.9)	-	-	WHO	EWGSOP2	Osteopenia/Ost-eoporosis + SP	-
Chew (2020) [54]	Asia	Cross- sectional	Community-dwelling	67.2 ± 7.4	23.9 ± 3.2	230	63	167	27 (11.7)	12 (19)	15 (8.9)	WHO	AWGS2	Osteoporosis + SP	-
Sepúlveda-Loyola (2020)^a^ [55]	Australia	Cross- sectional	Outpatient	77.9 ± 0.4	28.3 ± 6.0	253	57	196	48 (20.5) 25 (10.6) 47 (20.1)	-	-	WHO	EWGSOP2 EWGSOP FNIH	Osteopenia/Ost-eoporosis + SP	-
Okayama (2022) [56]	Japan	Cross- sectional	Outpatient	77.6 ± 8.1	22.4 ± 3.1	61	-	61	24 (39.3)	-	24 (39.3)	JOS	AWGS2	Osteoporosis + SP	-
Mathieu (2021) [57]	France	Cross- sectional	Hospitalized patients	84.8 ± 4.9	24.8 ± 5.6	101	-	101	33 (32.7)	-	33 (32.7)	French guidelines	EWGSOP2	Osteopenia/Ost-eoporosis + SP	-
Monaco (2020) [58]	Italy	Cross- sectional	Hospitalized patients	79.7 ± 7.2	-	350	-	350	230 (65.7)	-	230 (65.7)	WHO	FNIH	Osteoporosis + SP	-
Suriyaarachchi (2018) [59]	Australia	Cross- sectional	Outpatient	-	27.9 ± 6.1	400	140	260	160 (40.0)	28 (20)	132 (50.7)	WHO	EWGSOP2	Osteopenia/Ost-eoporosis + SP	Age (1.08, 1.0-1.1), Female (7.48, 3.9–14.0), Hyperparathyroidism (6.88, 1.9–9.2)
Buehring (2013) [60]	the United States	Cross- sectional	Community-dwelling	-	27.2 ± 4.8	304	146	158	33 (10.9)	-	-	WHO	Others	Osteoporosis + SP	-
Pechmann (2021) [61]	Brasil	Cross- sectional	Hospitalized patients	65.6 ± 8.6	29.2 ± 4.9	177	63	114	21 (11.9)	2 (3.1)	19 (10.7)	WHO	FNIH	Osteopenia/Ost-eoporosis + SP	-

WHO: World Health Organization; JOS: Japan Osteoporosis Society; AWGS, Asian Working Group for Sarcopenia; JSH: Japan Society of Hepatology; EWGSOP: European Working Group on Sarcopenia in Older People; FNIH: US Foundation for the National Institute of Health; CCS: Copenhagen Sarcopenia Study; ^a^: Consists of different diagnostic criteria of sarcopenia; SP: sarcopenia; PTH: parathyroid hormone; BMI, body mass index; IGF-1, insulin-like growth factor 1; *OR*: Odds Ratio; *CI*: Confidence Interval

### Quality of evidence

Among the 31 studies, 29 of them were cross-sectional studies. These studies were evaluated using the AHRQ scale. The final scores ranged between 4 and 7, indicating medium-quality literature (Additional file 1). Due to the nature of the study design, these studies did not explain how missing data were handled. There was also a lack of data integrity and follow-up results, thus all the cross-sectional studies failed to score on the three criteria: 9. “If applicable, explain how missing data were handled in the analysis”; 10. “Summarize patient response rates and completeness of data collection”; and 11. “Clarify what follow-up, if any, was expected and the percentage of patients for which incomplete data or follow-up was obtained”. As for the two cohort studies, the descriptions in comparability and follow-up time were clearer, thus resulting in quality scores ≥ 7, indicating them as high-quality literature (Additional file 2).

### Prevalence of osteosarcopenia and subgroup analysis

**Fig. 2:**
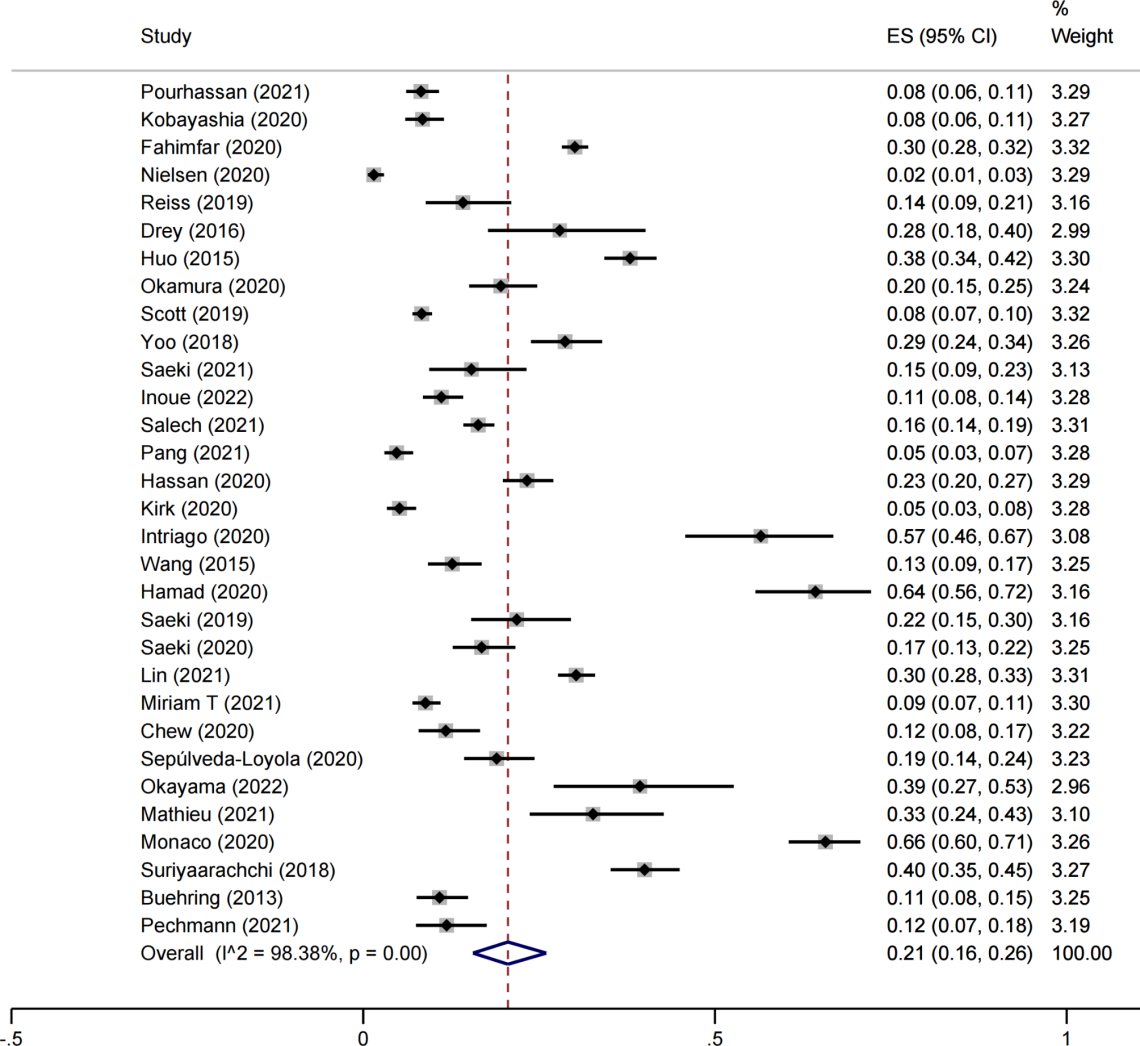
Meta-analysis of the prevalence of osteosarcopenia

The results showed that the prevalence of osteosarcopenia ranged from 1.5 to 65.7%, with an overall prevalence of 21% [95% *CI* 0.16–0.26)] (Fig. 2). A high heterogeneity between the studies was detected (*I*^2^ = 98.38%, *p* < 0. 05) using a random effects model.

To explore the sources of heterogeneity, publication distribution, gender, region, study population, diagnostic criteria for sarcopenia and definition of osteosarcopenia were used as subgroup analyses. Five studies from 2013 to 2017 showed that the prevalence of osteosarcopenia was 23% (95% *CI* 12–36%; *I*^2^ = 96.92, *p* = 0.00) while the prevalence of 26 studies from 2018 to 2022 was 20% (95% *CI* 15–26%; *I*^2^ = 98.50, *p* = 0.00). In terms of gender, the prevalence of osteosarcopenia among females was higher at 28% (95% *CI* 21–35%; *I*^2^ = 97.62, *p* = 0.00) than males at 14% (95% *CI* 9–20%; *I*^2^ = 95.25, *p* = 0.00). When analyzed by region, the prevalence was 18% (95% *CI* 13–24%; *I*^2^ = 97.16, *p* = 0.00) in 13 studies from Asia while the eight European studies showed a prevalence of 26% (95% *CI* 11–45%; *I*^2^ = 99.09, *p* = 0.00). The prevalence in the six Oceania studies was 21% (95% *CI* 10–34%; *I*^2^ = 98.91, *p* = 0.00) while the three South American studies showed a prevalence of 23% (95% *CI*: 5–48%) as compared to the study from North America with a prevalence of 11% (95% *CI*: 8–15%) (Additional file 3).

According to the grouping of the study population, the prevalence of osteosarcopenia was higher among hospitalized and outpatient participants with a prevalence of 26% (95%*CI* 18–36%; *I*^2^ = 97.72, *p* = 0.00) and 33% (95% *CI* 16–53%; *I*^2^ = 98.09, *p* = 0.00) respectively. The prevalence was much lower in the community-dwelling group with 12% (95% *CI* 7–18%; *I*^2^ = 98.37, *p* = 0.00). Based on the different diagnostic criteria of sarcopenia, the prevalence was 15% under the EWGSOP2 criteria (95% *CI* 7–25%; *I*^2^ = 98.33, *p* = 0.00) as compared to 30% (95% *CI* 19–42%; *I*^2^ = 98.82, *p* = 0.00) when using the EWGSOP criteria. When using AWGS2 and JSH criteria, the prevalence of osteosarcopenia was 17% (95% *CI* 7–31%; *I*^2^ = 98.23, *p* = 0.00) and 18% (95% *CI*: 15–21%), respectively. When using AWGS criteria, the prevalence was 20% (95% *CI*: 11–30%). When using the FNIH criteria, the prevalence was higher at 46% (95% *CI*: 42–50%). The prevalence of osteosarcopenia under other criteria was the lowest at 9% (95% *CI*: 7–12%). When using sarcopenia plus osteoporosis to define osteosarcopenia, its prevalence is 17% (95% *CI*: 10–26%), but when using another definition, the prevalence increases to 24% (95% *CI*: 17–32%). (Table 2) (Additional file 4).

**Table 2:** Subgroup analysis of the prevalence of osteosarcopenia based on various factors

Subgroup	Number of trials	Heterogeneity test		Effect model	*ES* (95% *CI*)
		*I*^2^(%)	*P*-value		
Year					
2013–2017	5	96.92	0.00	random	0.23 (0.12–0.36)
2018–2022	26	98.50	0.00	random	0.20 (0.15–0.26)
Sex					
Female	23	97.62	0.00	random	0.28 (0.21–0.35)
Male	18	95.25	0.00	random	0.14 (0.09–0.20)
Region					
Europe	8	99.09	0.00	random	0.26 (0.11–0.45)
Asia	13	97.16	0.00	random	0.18 (0.13–0.24)
Oceania	6	98.91	0.00	random	0.21 (0.10–0.34)
South America	3	-	-		0.23 (0.05–0.48)
North America	1	-	-		0.11 (0.08–0.15)
Study population					
Hospitalized	13	97.72	0.00	random	0.26 (0.18–0.36)
Community-dwelling	13	98.37	0.00	random	0.12 (0.07–0.18)
Outpatient	5	98.09	0.00	random	0.33 (0.16–0.53)
Diagnostic criteria of sarcopenia					
EWGSOP2	8	98.33	0.00	random	0.15 (0.07–0.25)
EWGSOP	8	98.82	0.00	random	0.30 (0.19–0.42)
AWGS2	5	98.23	0.00	random	0.17 (0.07–0.31)
AWGS	3	-	-	-	0.20 (0.11–0.30)
JSH	3	-	-	-	0.18 (0.15–0.21)
FNIH	2	-	-	-	0.46 (0.42–0.50)
Others	2	-	-	-	0.09 (0.07–0.12)
Definition of osteosarcopenia					
Osteopenia/Osteoporosis + SP	16	98.55	0.00	random	0.24 (0.17–0.32)
Osteoporosis + SP	15	98.07	0.00	random	0.17 (0.10–0.26)

EWGSOP: European Working Group on Sarcopenia in Older People; AWGS, Asian Working Group for Sarcopenia; JSH: Japan Society of Hepatology; FNIH: US Foundation for the National Institute of Health; SP: sarcopenia

### Sensitivity analysis and publication bias

The results of sensitivity analysis showed that with the exclusion of any one literature, the combined prevalence did not change significantly, thus indicating good stability of the meta-analysis results (Additional file 5). The funnel plot scatter was not uniform and symmetrical in Additional file 6, so the publication bias was further tested using *Egger*’s test and *Begg*’s test (*t* = 0.46, *p* = 0.650; *z* = 1.94, *p* = 0.053). From the results, it could be considered that there was no publication bias (Additional file 7).

### Risk factors of osteosarcopenia

**Fig. 3:**
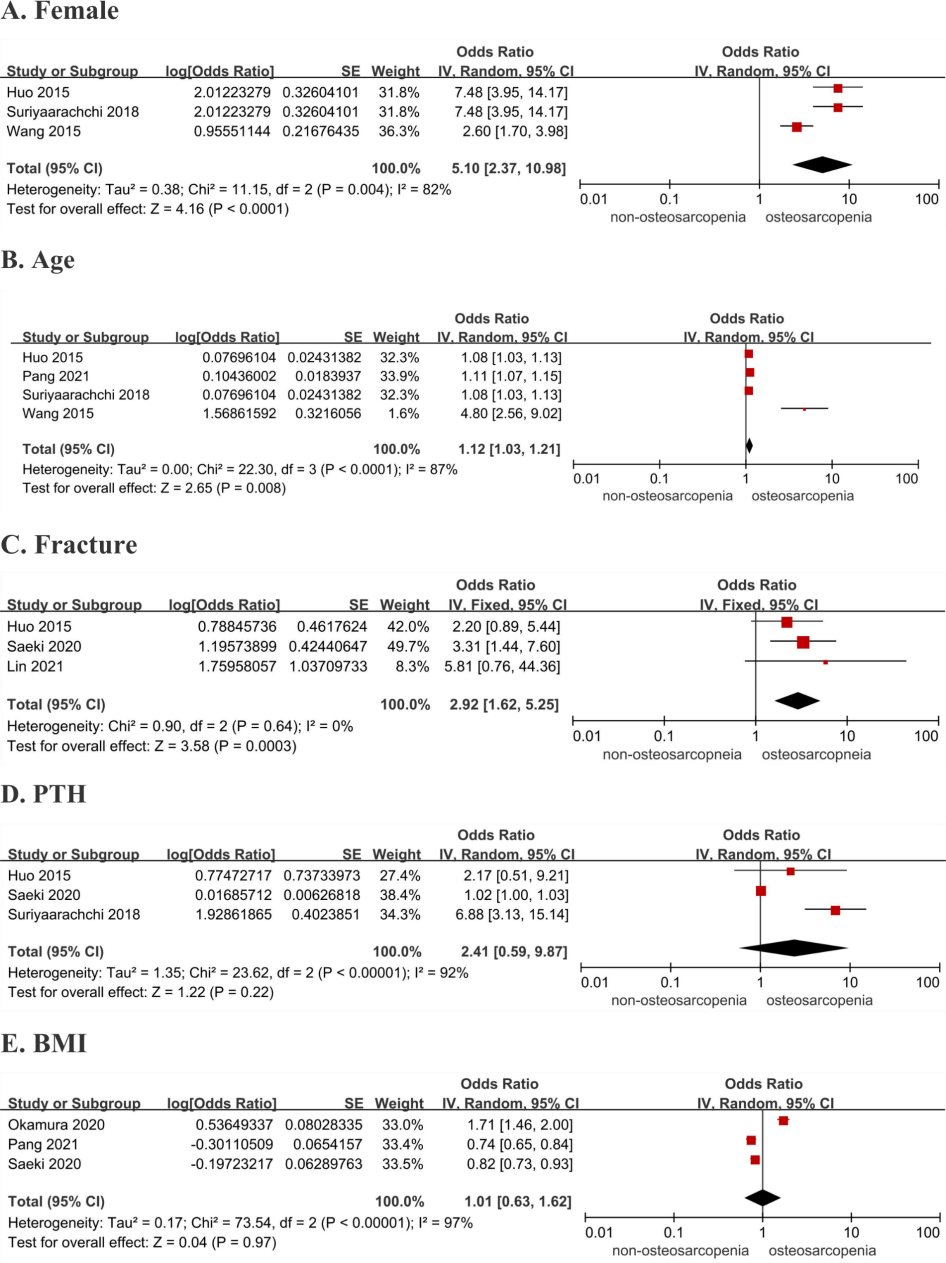
Forest plots of pooled *OR*s for various risk factors. As shown in the figure, data presented as *OR*s with their respectively 95% *CI*s. *I*^2^ was uesd to assess the heterogeneity, selecting the appropriate effect model according to the heterogeneity. Using the data presented in the selected articles, the final search for risk factors for osteosarcopenia was made from five factors (respectively, female, age, fracture, PTH and BMI)

The results showed that females (*OR* = 5.10, 95% *CI* 2.37–10.98, *p* < 0.0001), older age (*OR* = 1.12, 95% *CI* 1.03–1.21, *p* = 0.008), and fracture (*OR* = 2.92, 95% *CI*: 1.62–5.25, *p* = 0.0003) were risk factors of osteosarcopenia. However, high parathyroid hormone (PTH) (*OR* = 2.41, 95% *CI*: 0.59–9.87, *p* = 0.22) and high body mass index (BMI) (*OR* = 1.01, 95% *CI* 0.63–1.62, *p* = 0.97) were not significantly associated with osteosarcopenia. Meta-analysis could not be performed for other factors due to insufficient data (Fig. 3) (Additional file 8).

## Discussion

This paper comprehensively reviewed studies to obtain a pooled prevalence of osteosarcopenia and its risk factors. The final results showed that the prevalence of osteosarcopenia was 21% and females, old age, and a history of fractures were significant risk factors for the condition.

Osteosarcopenia is a new geriatric syndrome that describes the coexistence of osteopenia/osteoporosis with sarcopenia [1, 19, 62]. The function of muscles and bones are closely related, and studies have shown that many environmental factors (such as lack of exercise, poor nutrition, obesity, aging, and gender) [63, 64] can lead to muscle and bone loss through the interaction of genetic, mechanical,and endocrine factors [18, 64]. In addition, studies have established an association between osteosarcopenia and the risk of frailty, falls, and fractures, as well as with non-communicable diseases [28]. In a Chilean study, 16.4% of elderly aged 60 and older living in the community had osteosarcopenia [25]. In a cross-sectional study of 142 patients with liver cirrhosis, the prevalence of osteosarcopenia was 21.8% [50]. Apart from pharmacological treatment, most of the research has also focused on non-pharmacological methods, including exercise, particularly resistance training, and nutritional support (supplementing with protein, vitamin D, and calcium) [65]. Studies have shown that exercising 2–3 times a week for at least 20 min can significantly improve muscle and bone density [1]. To maximize muscle and skeletal health, it is also necessary to meet dietary recommendations: protein (1.2–1.5 g/kg/day), vitamin D (800–1000 IU/day), calcium (1300 mg/day), and creatine (3–5 g/day) [64]. In the context of effective interventions, an increasing number of scholars propose the necessity of a multidisciplinary comprehensive management approach. The identification and assessment of osteosarcopenia are particularly important for preventing adverse health outcomes, including detailed medical history inquiries, risk factor identification, and physical evaluations [64, 66, 67]. First, it is necessary to inquire about relevant medical histories,such as age, medical history, falling history, fracture history, and medication use, in order to identify disease risk. For individuals with increased risk, sarcopenia and osteoporosis evaluations can be conducted using risk assessment tools such as the Red Flag Method, the SARC-F questionnaire [68], or the skeletal muscle index (SMI) method [69], as well as a fracture risk assessment tool (FRAX) [70]. In addition, inadequate protein intake can significantly affect bone health [66], and The Mini-Nutritional Assessment should also be considered to assess the risk of malnutrition easily and quickly [71]. A thorough physical evaluation includes measurement of muscle strength (grip strength, the repeated chair stand test), muscle mass [69], physical performance (gait speed, short physical performance battery), and bone density. It is recommended to evaluate muscle mass and bone density mainly through Dual-energy x-ray absorptiometry (DXA) [72]. If DXA is not available computed tomography (CT), magnetic resonance imaging (MRI) or bio-electrical impedance analysis (BIA) can also be used to evaluate muscle mass [72]; vertebral imaging or bone turnover markers(BTMs) can be used for osteoporosis assessment [73]. However, these methods are less accurate than DXA.

Subgroup analyses were performed to explore the sources of heterogeneity. There were significant differences in the prevalence of osteosarcopenia in terms of year of publication, gender, region, study population, and diagnostic criteria. For instance, the prevalence in 2018–2022 was lower than in 2013–2017. A cross-sectional study in China found that malnutrition and frailty were highly prevalent among elderly hospitalized patients in which malnutrition was associated with an increased risk of frailty [74]. As living conditions continue to improve, the nutritional status among the general population is now better than before, thus likely attributing to a lower prevalence of malnutrition and its associated comorbidities. In the review, the results also showed that inpatient and outpatient participants were more likely to develop osteosarcopenia than those living in the community. Similarly, previous studies have highlighted that hospitalized older adults and those living in nursing homes were particularly susceptible to muscle-related diseases [75, 76]. The outpatient participants included in this study were seen in specialty clinics such as osteoporosis clinics, falls and fracture clinics, or frailty clinics, thus putting them in the high-risk population for the disease. Furthermore, some studies reported that non-Asian populations appeared to be more susceptible to sarcopenia [76]. One possible reason could be the differences in ethnic characteristics, body size, and dietary regimes. Nevertheless, the prevalence obtained in this review was higher among Asian populations than non-Asian populations, except for North America. The inclusion of only one study from North America is likely not sufficient to represent the prevalence in the general elderly population.

Overall, the prevalence of osteosarcopenia among females was higher (25.5–82.6%) compared to men [36, 47]. The changes in estrogen levels in females can affect the functions of bones and muscles. Firstly, estrogen inhibits bone turnover and prevents bone loss [77]. It also affects skeletal muscle by increasing the level of inflammatory factors in the skeletal muscle environment by resisting proteolysis as well as promoting the proliferation and differentiation of muscle satellite cells [78]. Compared to men, women are at a higher risk because their weight and BMD are commonly lower than men of the same age [79, 80]. With aging, the expression of vitamin D receptors on the cell membranes of skeletal muscle fiber decreases, further exacerbating vitamin D deficiency in the elderly [81]. In addition, aging can lead to a loss of muscle strength and mass, subsequently changing the skeletal microstructure and decreasing the mineral density, resulting in decreased bone mass [82]. All these factors predispose to fractures. Among the elderly, fractures can compromise physical activity function [83, 84] and quality of life. Therefore, it is essential to maintain BMD, muscle strength, as well as bone and muscle mass [84]. Fractures may lead to further loss of muscle and bone mass due to immobility, thus predisposing the patients to a higher risk of osteosarcopenia. In short, the pathogenesis of osteosarcopenia is closely related to the interaction between multiple endocrine, nutritional, genetic, and lifestyle factors [85].

There are several advantages to the current study. Firstly, it is a large global sample that quantitatively combines the prevalence and risk factors of osteosarcopenia. Secondly, through subgroup analysis (publication distribution, gender, region, study population, diagnostic criteria for sarcopenia, definition of osteosarcopenia), heterogeneous factors that lead to the estimated prevalence of osteosarcopenia reduction are explored. Finally, this study proposes strategies for preventing and managing osteosarcopenia, such as dietary regulation, exercise intervention, medication therapy, physical assessment, etc., providing clinical doctors with effective guidance and suggestions. Overall, this study has practicality and operability, and provides important references for the health management of the elderly.

This study also has some shortcomings. Firstly, This systematic review does not include any articles not written in English. Next, the heterogeneity of the combined data in the selected studies was high. Even after subgroup analysis, the heterogeneity between studies was still high. If sufficient data are available, severe sarcopenia can be evaluated separately in the diagnosis. Additionally, some studies measured only muscle mass and strength without any assessment of step speed. Thus, this could have influenced the overall results. In addition, we could not summarize the prevalence by age groups as different age groups were used by the studies. For instance, one study reported the prevalence of osteosarcopenia for the age groups of 65–74, 75–84, and ≥ 85 years old [40]while patients in another study from Chile was grouped into 60–69, 70–79, and ≥ 80 years old [25]. Most of the included cross-sectional studies had large differences in sample size, thus leading to more confounding factors and greater heterogeneity. Therefore, the strength of the argument was likely insufficient. More high-quality prospective studies are recommended to verify the review findings.

## Conclusions

In summary, the meta-analysis found that the prevalence of osteosarcopenia was high. Moreover, females, advanced age, and a history of fracture were independently associated with osteosarcopenia. Therefore, early assessment and timely intervention should be undertaken among high-risk populations to prevent or delay the disease progression.

## Electronic supplementary material

Below is the link to the electronic supplementary material.

Supplementary Material 1

Supplementary Material 2

Supplementary Material 3

Supplementary Material 4

Supplementary Material 5

Supplementary Material 6

Supplementary Material 7

Supplementary Material 8

Supplementary Material 9

## Data Availability

The datasets used and/or analysed during the current study are available from the corresponding author on reasonable request.

## List of abbreviations

AHRQ: US Agency for Healthcare Quality and Research
AWGS: Asian Working Group for Sarcopenia
BIA: Bio-electrical impedance analysis
BMD: Bone mineral density
BMI: Body mass index
BTMs: Bone turnover markers
CBM: China Biomedical Literature Database
CCS: Copenhagen Sarcopenia Study
*CI*: Confidence Interval
CNKI: China National Knowledge Infrastructure
CT: Computed tomography
DXA: Dual-energy x-ray absorptiometry
EWGSOP: European Working Group on Sarcopenia in Older People
FNIH: US Foundation for the National Institutes of Health
FRAX: A fracture risk assessment tool
GDF8: Growth/differentiation factor 8
GLYAT: Glycine-N-acyltransferase
GWAS: Genome-wide association studies
IGF-1: Insulin-like growth factor 1
IWGS: International Working Group for Sarcopenia
JOS: Japan Osteoporosis Society
JSH: Japan Society of Hepatology
MEF2C: Myocyte enhancer factor-2 C
METTL21C: Methyltransferase-like 21 C
MRI: Magnetic resonance imaging
NOS: Newcastle-Ottawa Scale
OS: Osteosarcopenia
*OR*: Odds Ratio
PCOS: Polycystic ovary syndrome
PGC-1α: Peroxisome proliferator-activated receptor gamma coactivator 1-alpha
PRISMA: Preferred Reporting Items for Systematic Review and Meta-Analysis
PTH: Parathyroid hormone
SD: Standard deviation
SMI: Skeletal muscle index
SREBF1: Sterol regulatory element-binding transcription factor 1
STATA: Stata Corporation, College Station
WHO: World Health Organization
