# Supplementary material for: Prevalence and risk factors of osteosarcopenia: a systematic review and meta-analysis
Source: BMC Geriatr. 2023 Jun 15;23:369. doi: 10.1186/s12877-023-04085-9 (PMC10273636; doi:10.1186/s12877-023-04085-9)
Supplement: Supplementary file 1 — Supplementary Material 1 [file 12877_2023_4085_MOESM1_ESM.doc]

supplement Table 1. Assessment of the quality of cross-sectional studies based on the criteria recommended by the US Agency for Healthcare Quality and Research (AHRQ).

| Study | Q1 | Q2 | Q3 | Q4 | Q5 | Q6 | Q7 | Q8 | Q9 | Q10 | Q11 |
| --- | --- | --- | --- | --- | --- | --- | --- | --- | --- | --- | --- |
| Pourhassan 2021 | Yes | Yes | Yes | Yes | Unclear | Yes | Unclear | Yes | No | No | No |
| Kobayashia 2020 | Yes | Yes | Yes | Yes | Unclear | Yes | Unclear | Unclear | No | No | No |
| Fahimfar 2020 | Yes | Yes | Yes | Yes | Unclear | Yes | Unclear | Yes | No | No | No |
| Nielsen 2020 | Yes | Yes | No | Yes | Unclear | Yes | Unclear | Yes | No | No | No |
| Reiss 2019 | Yes | Yes | No | Yes | Unclear | Yes | Unclear | Yes | No | No | No |
| Drey 2016 | Yes | Yes | No | No | Unclear | Yes | No | Yes | No | No | No |
| Huo 2015 | Yes | Yes | Yes | Yes | Unclear | Yes | No | Yes | No | No | No |
| Okamura 2020 | Yes | Yes | Yes | Yes | Unclear | Yes | Yes | Yes | No | No | No |
| Saeki 2021 | Yes | Yes | Yes | Yes | Unclear | Yes | No | Yes | No | No | No |
| Inoue 2022 | Yes | Yes | Yes | Yes | Unclear | Yes | Unclear | Yes | No | No | No |
| Salech 2021 | Yes | Yes | No | Yes | Unclear | Yes | Unclear | Yes | No | No | No |
| Pang 2021 | Yes | Yes | Yes | No | Unclear | Yes | No | Yes | No | No | No |
| Hassan 2020 | Yes | Yes | Yes | Yes | No | Yes | No | Yes | No | No | No |
| Kirk 2020 | Yes | Yes | No | Yes | Unclear | Yes | No | Yes | No | No | No |
| Intriago 2020 | Yes | Yes | Yes | Yes | Unclear | Yes | No | No | No | No | No |
| Wang 2015 | Yes | Yes | Yes | Yes | Unclear | Yes | No | Yes | No | No | No |
| Hamad 2020 | Yes | Yes | Yes | Yes | Unclear | Yes | Unclear | Unclear | No | No | No |
| Saeki 2019 | Yes | Yes | Yes | Yes | Unclear | Yes | Yes | Yes | No | No | No |
| Saeki 2020 | Yes | Yes | Yes | Yes | Unclear | Yes | No | Yes | No | No | No |
| Lin 2021 | Yes | Yes | Yes | Yes | Unclear | Yes | No | Yes | No | No | No |
| Miriam T 2021 | Yes | Yes | No | Yes | No | Yes | Yes | Yes | No | No | No |
| Chew 2020 | Yes | Yes | No | Yes | Unclear | Yes | No | Yes | No | No | No |
| Sepúlveda-Loyola 2020 | Yes | Yes | Yes | Yes | Unclear | Yes | Unclear | Yes | No | No | No |
| Okayama 2022 | Yes | Yes | Yes | Yes | Unclear | Yes | Unclear | Yes | No | No | No |
| Mathieu 2021 | Yes | Yes | Yes | Yes | Unclear | Yes | Unclear | Yes | No | No | No |
| Monaco 2020 | Yes | Yes | No | Yes | Unclear | Yes | Yes | Yes | No | No | No |
| Suriyaarachchi 2018 | Yes | Yes | Yes | Yes | Unclear | Yes | No | Yes | No | No | No |
| Buehring 2013 | Yes | Yes | Yes | Yes | Unclear | Yes | No | No | No | No | No |
| Pechmann 2021 | Yes | Yes | Yes | Yes | Unclear | Yes | Yes | Yes | No | No | No |

Q1: Define the source of information (survey, record review)

Q2: List the inclusion and exclusion criteria for exposed and unexposed subjects (cases and controls) or refer to previous publications

Q3: Indicate time period used for identifying patients

Q4: Indicate whether or not subjects were consecutive if not population-based

Q5: Indicate if evaluators of subjective components of study were masked to other aspects of the status of the participants

Q6: Describe any assessments undertaken for quality assurance purposes (e.g., test/retest of primary outcome measurements)

Q7: Explain any patient exclusion from analysis

Q8: Describe how confounder was assessed and/or controlled

Q9: If applicable, explain how missing data were handled in the analysis

Q10: Summarize patient response rates and completeness of data collection

Q11: Clarify what follow-up, if any, was expected and the percentage of patients for which incomplete data or follow-up was obtained

(Yes/No/Unclear)
