# Supplementary material for: Prevalence and risk factors of osteosarcopenia: a systematic review and meta-analysis
Source: BMC Geriatr. 2023 Jun 15;23:369. doi: 10.1186/s12877-023-04085-9 (PMC10273636; doi:10.1186/s12877-023-04085-9)
Supplement: Supplementary file 2 — Supplementary Material 2 [file 12877_2023_4085_MOESM2_ESM.doc]

supplement Table 2. Quality of included studies based on the Newcastle–Ottawa scale.

| Studies | Selection  (0–4 stars) | | | | Comparability  (0–2 stars) | Outcome  (0–3stars) | | | Total NOS  score (0–9) |
| --- | --- | --- | --- | --- | --- | --- | --- | --- | --- |
|  | Q1 | Q2 | Q3 | Q4 | Q5 | Q6 | Q7 | Q8 |  |
| Scott 2019 | * | * | * | * | * | * | * | * | 8 |
| Yoo 2018 | * | * | * | - | * | * | * | * | 7 |
| Q1: Representativeness of the exposed cohort | | | | | | | | | |
| Q2: Selection of the non-exposed cohort | | | | | | | | | |
| Q3: Ascertainment of exposure | | | | | | | | | |
| Q4: Demonstration that outcome of interest was not present at the start of the study | | | | | | | | | |
| Q5: Comparability of cohorts on the basis of the design or analysis | | | | | | | | | |
| Q6: Assessment of outcome | | | | | | | | | |
| Q7: Was followed up long enough for outcomes to occur | | | | | | | | | |
| Q8: Adequacy of follow-up of cohorts | | | | | | | | | |

“*” represents one point
