# Supplementary material for: Prevalence and risk factors of osteosarcopenia: a systematic review and meta-analysis
Source: BMC Geriatr. 2023 Jun 15;23:369. doi: 10.1186/s12877-023-04085-9 (PMC10273636; doi:10.1186/s12877-023-04085-9)
Supplement: Supplementary file 3 — Supplementary Material 3 [file 12877_2023_4085_MOESM3_ESM.doc]

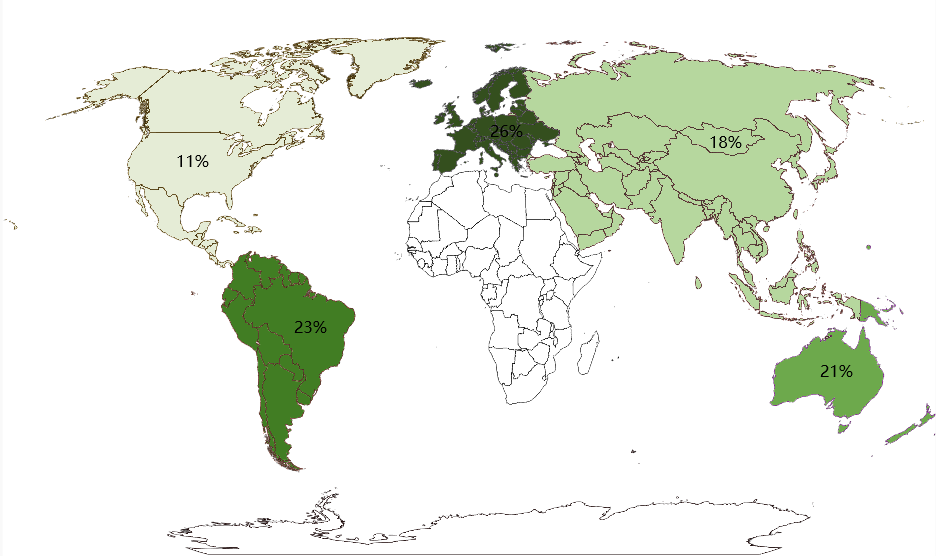


supplement Fig. 1. Prevalence of osteosarcopenia by region. Data are shown in the corresponding graphs for each region.
