# Supplementary material for: Prevalence and risk factors of osteosarcopenia: a systematic review and meta-analysis
Source: BMC Geriatr. 2023 Jun 15;23:369. doi: 10.1186/s12877-023-04085-9 (PMC10273636; doi:10.1186/s12877-023-04085-9)
Supplement: Supplementary file 4 — Supplementary Material 4 [file 12877_2023_4085_MOESM4_ESM.doc]

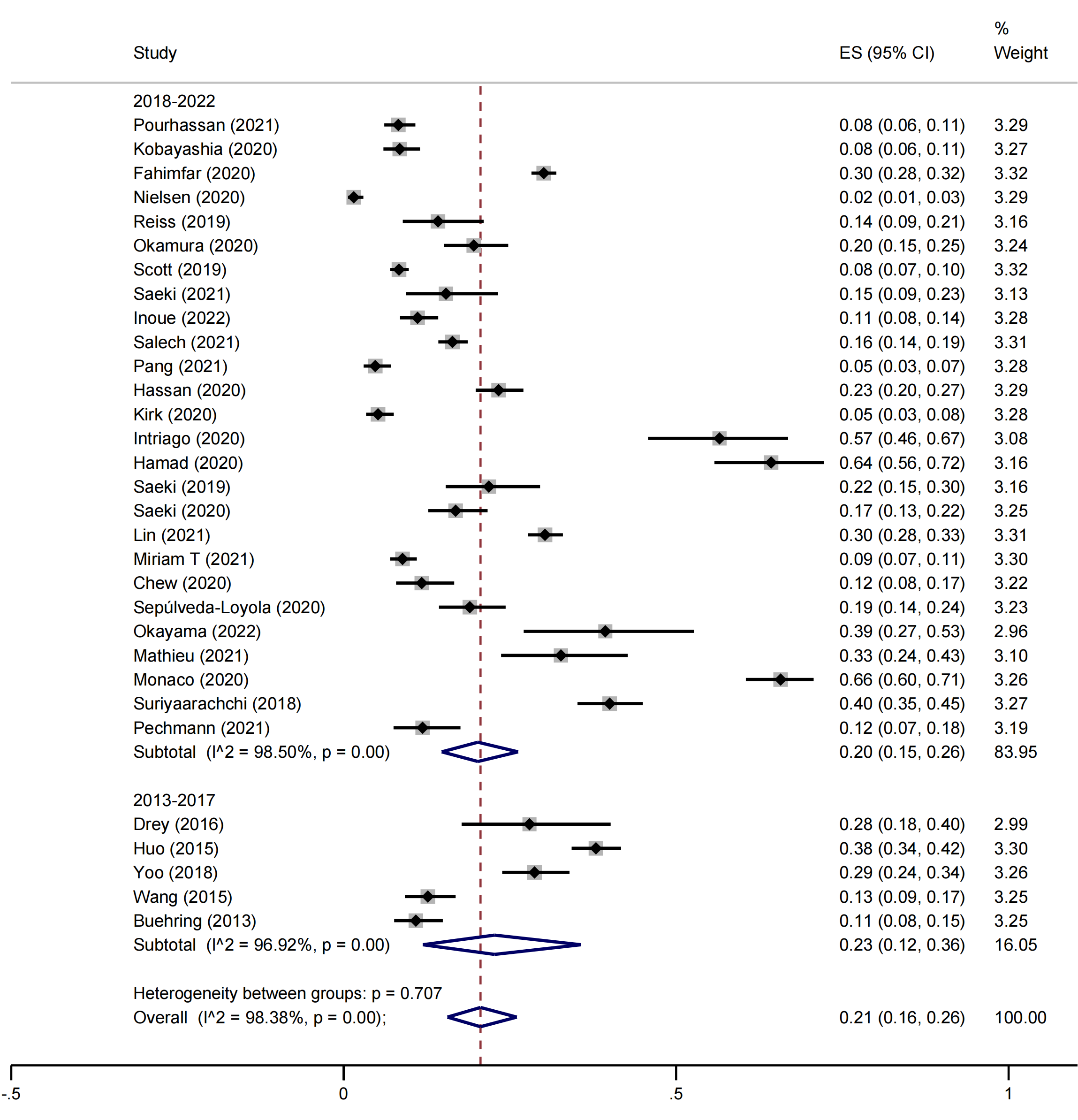


supplement Fig. 2a. Forest plot of the prevalence of oeteosarcopenia based on publication distribution.


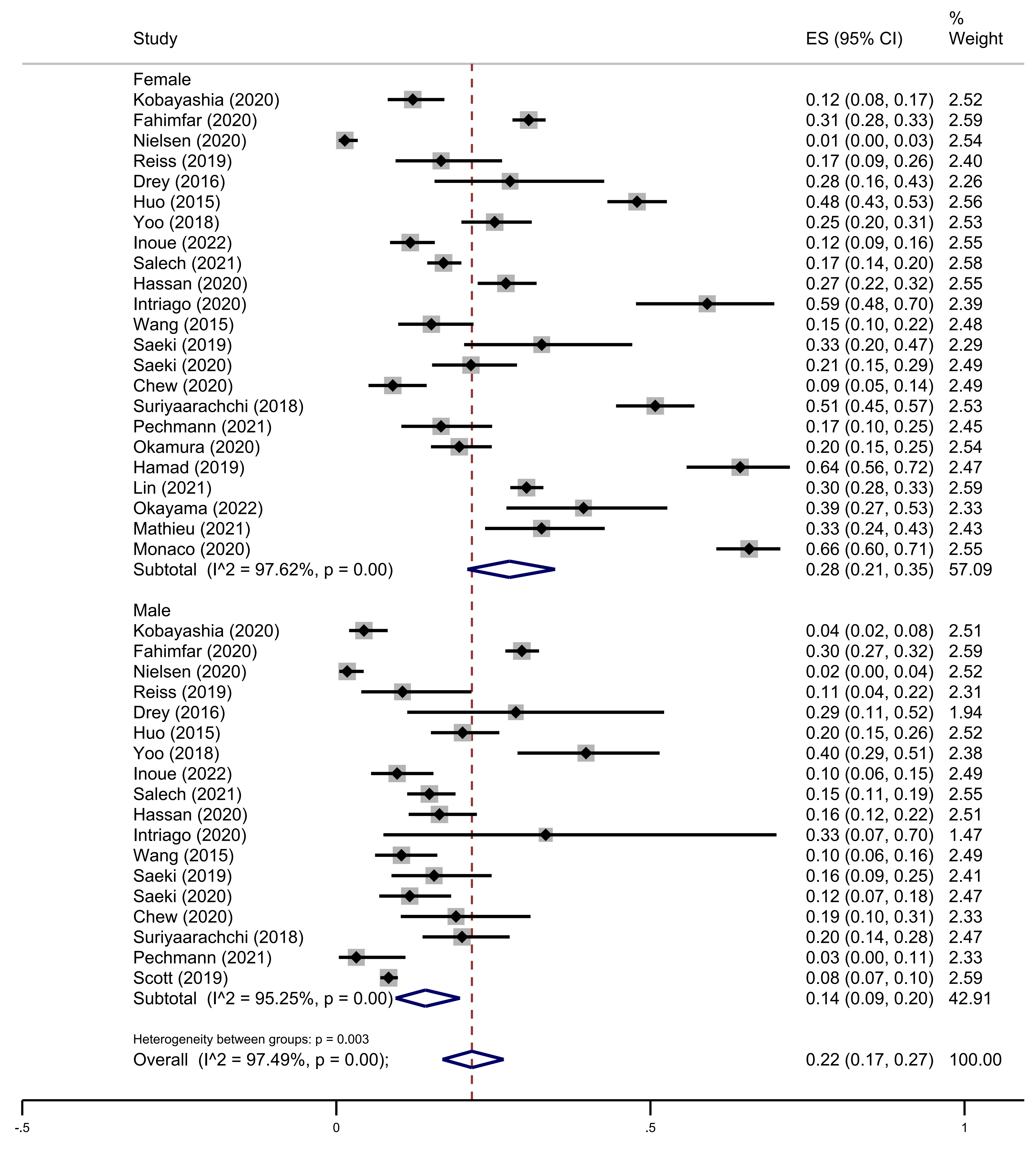


supplement Fig. 2b. Forest plot of the prevalence of oeteosarcopenia by gender.


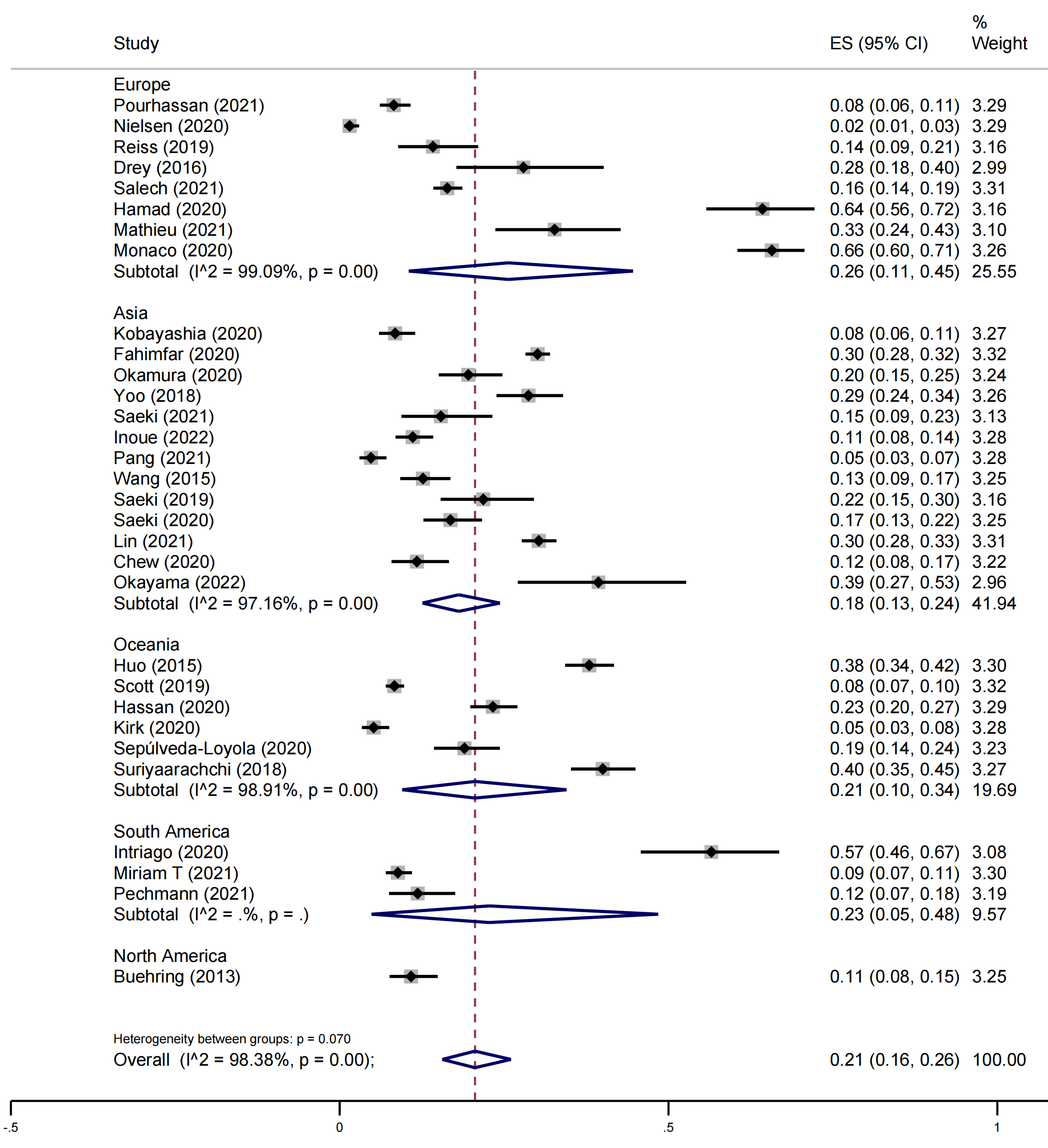


supplement Fig. 2c. Forest plot of the prevalence of oeteosarcopenia by region.


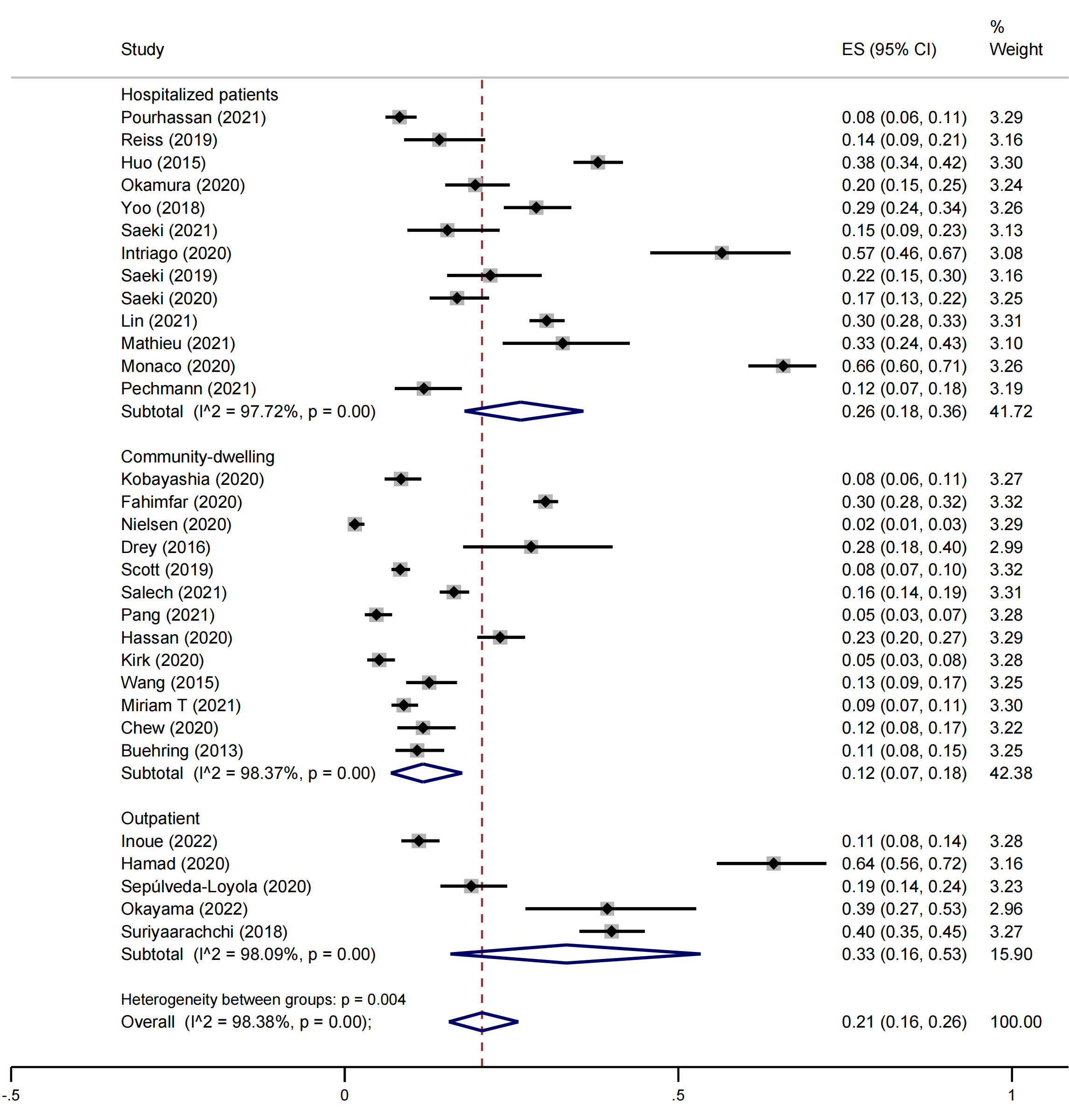


supplement Fig. 2d. Forest plot of the prevalence of oeteosarcopenia by study population.


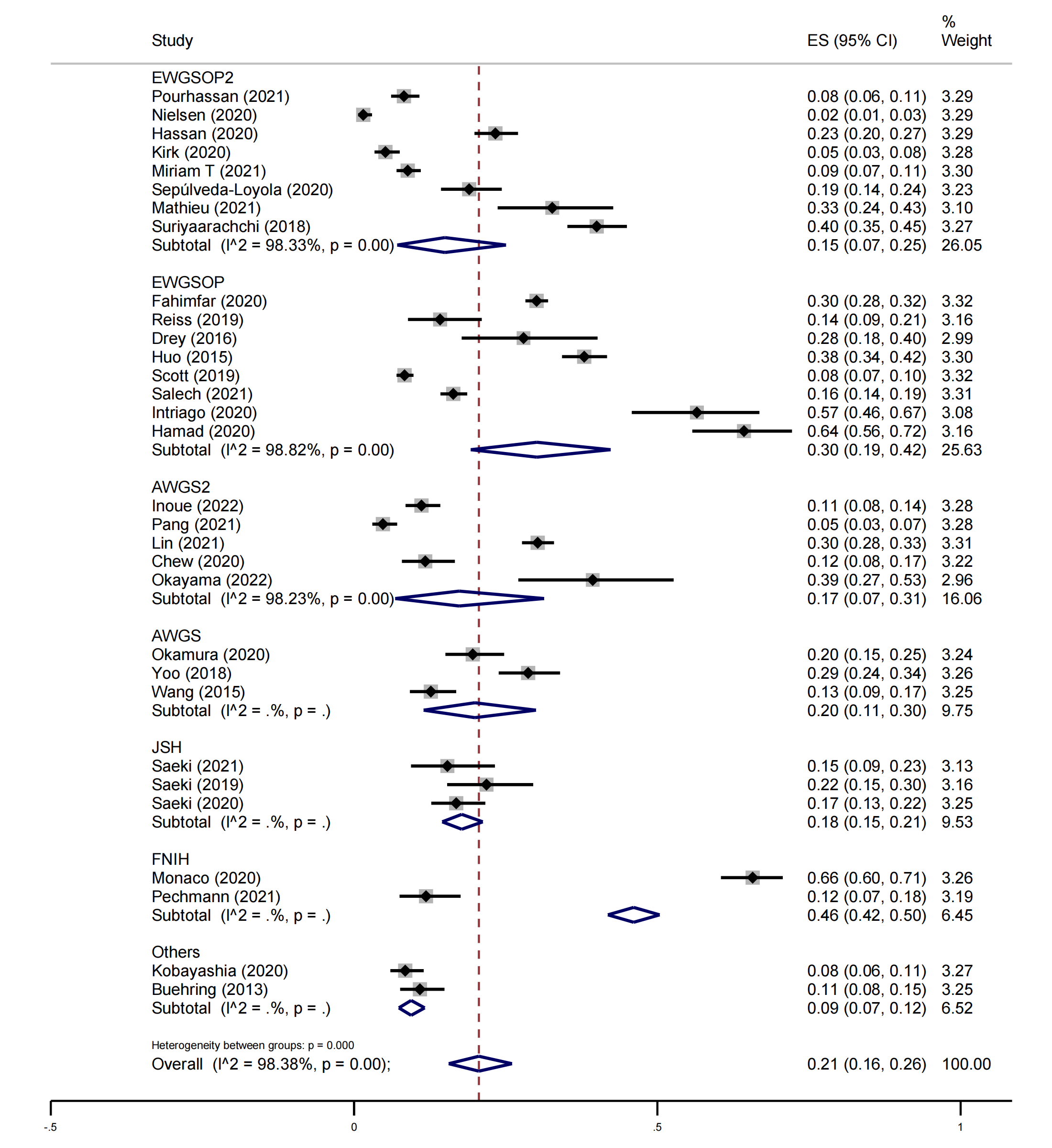


supplement Fig. 2e. Forest plot of the prevalence of oeteosarcopenia by the diagnostic criteria of sarcopenia.


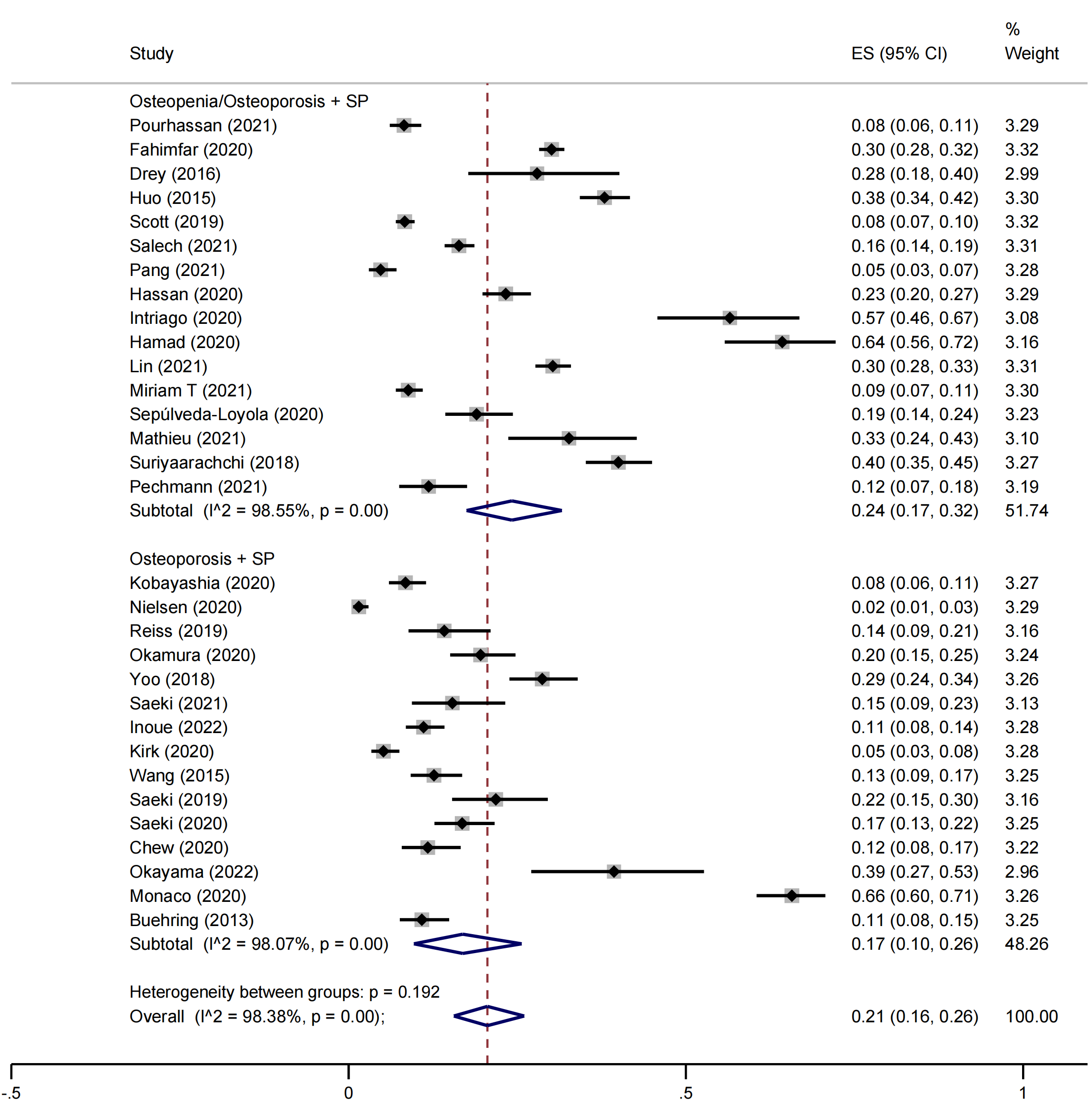


supplement Fig. 2f. Forest plot of the prevalence of oeteosarcopenia by the definition of osteosarcopenia.
