# Supplementary figures and images for: Prevalence and risk factors of osteosarcopenia: a systematic review and meta-analysis
Source: BMC Geriatr. 2023 Jun 15;23:369. doi: 10.1186/s12877-023-04085-9 (PMC10273636; doi:10.1186/s12877-023-04085-9)

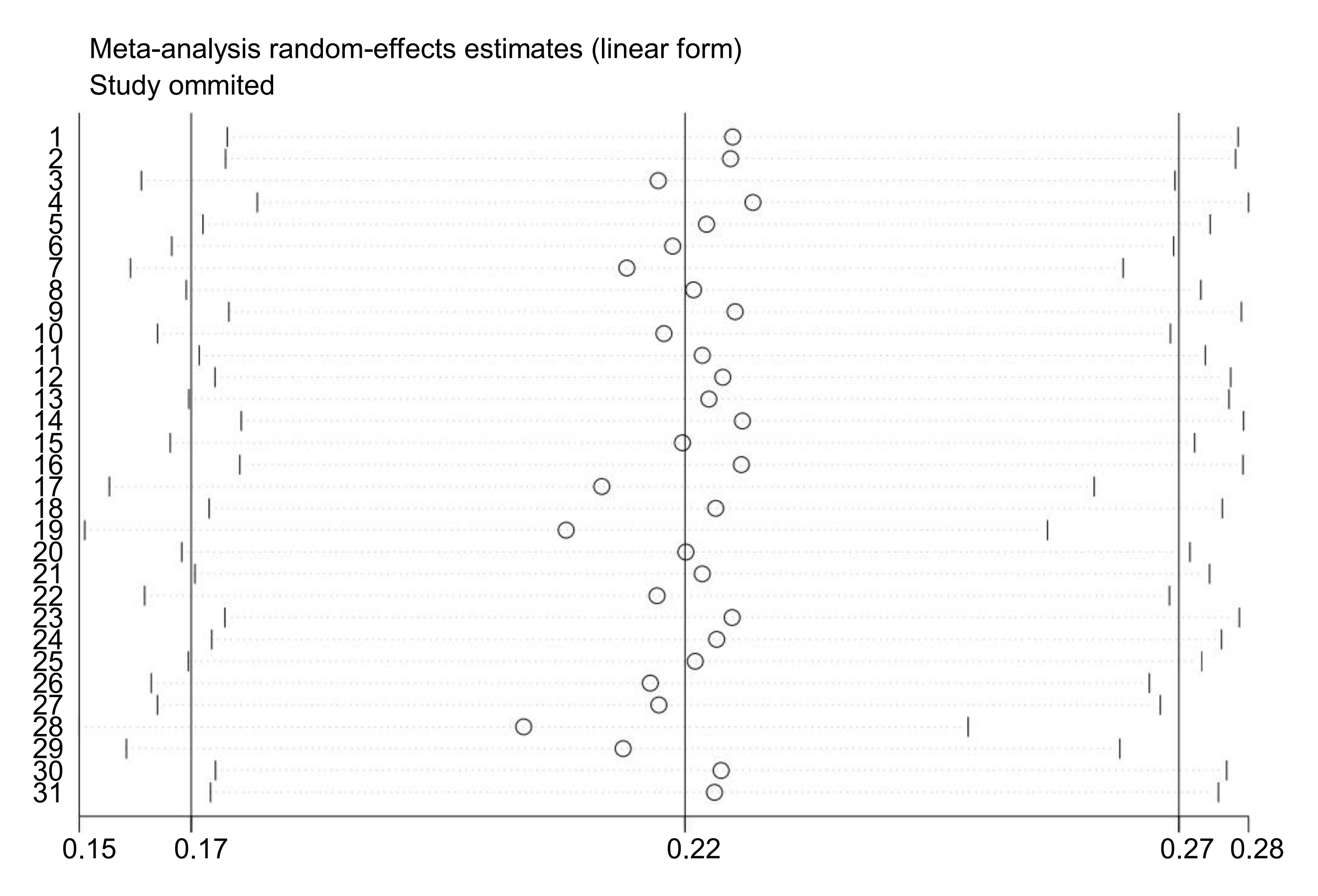


supplement Fig. 3. Sensitivity analysis of the prevalence of osteosarcopenia.

Supplement: Supplementary file 5 — Supplementary Material 5 [file 12877_2023_4085_MOESM5_ESM.doc]
