# Supplementary material for: Prevalence and risk factors of osteosarcopenia: a systematic review and meta-analysis
Source: BMC Geriatr. 2023 Jun 15;23:369. doi: 10.1186/s12877-023-04085-9 (PMC10273636; doi:10.1186/s12877-023-04085-9)
Supplement: Supplementary file 7 — Supplementary Material 7 [file 12877_2023_4085_MOESM7_ESM.doc]

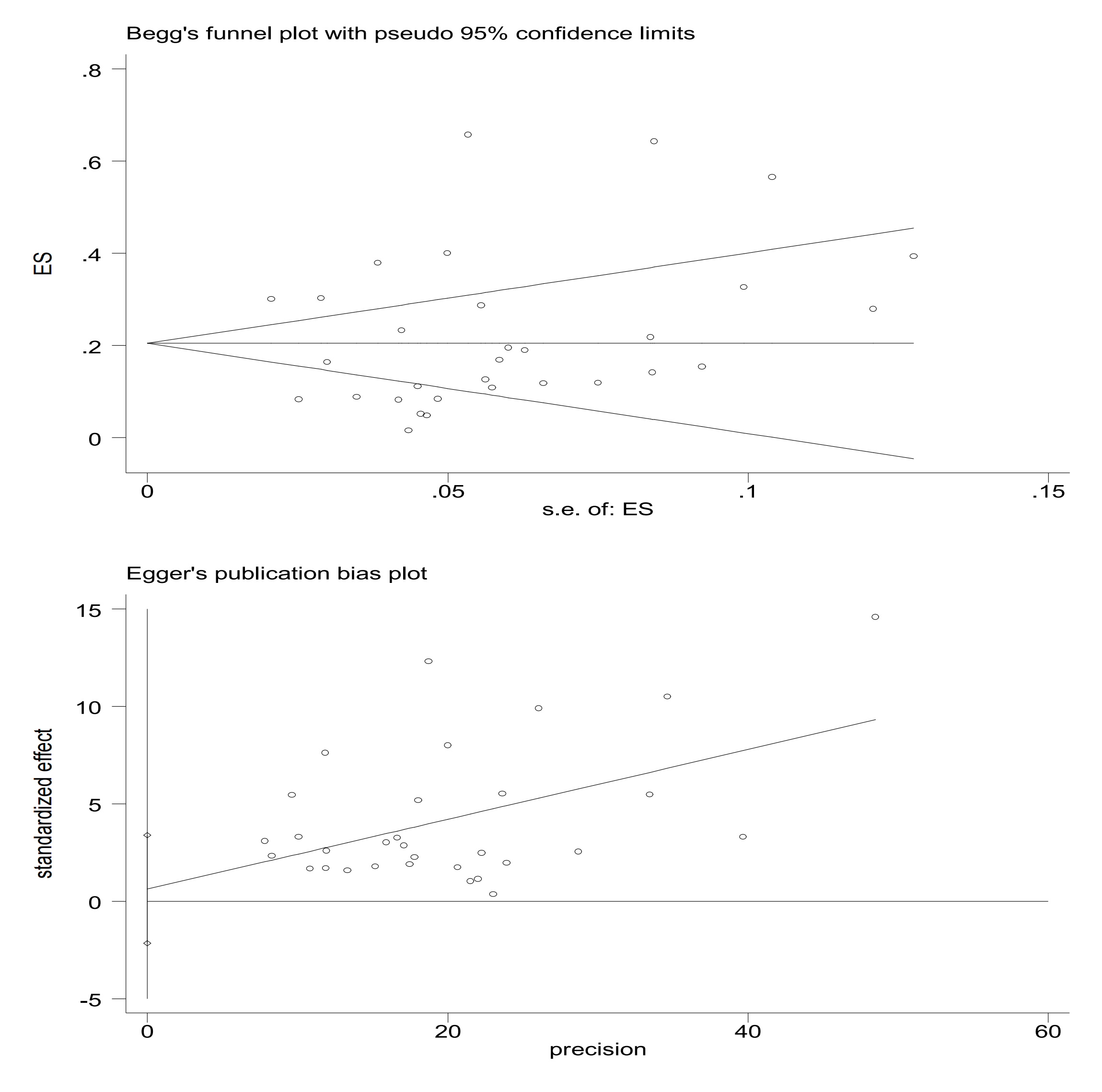


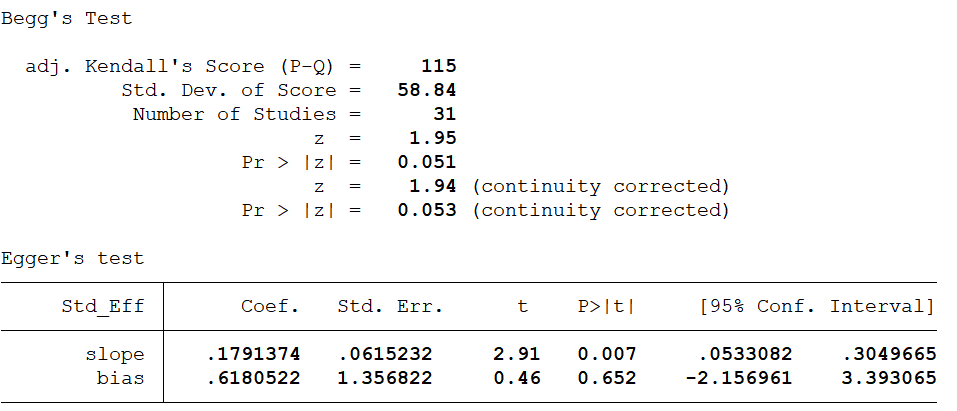


supplement Fig 5. Egger’s and Begg’s test of the risk of publication bias for the prevalence of osteosarcopenia.
