# Supplementary material for: Prevalence and risk factors of osteosarcopenia: a systematic review and meta-analysis
Source: BMC Geriatr. 2023 Jun 15;23:369. doi: 10.1186/s12877-023-04085-9 (PMC10273636; doi:10.1186/s12877-023-04085-9)
Supplement: Supplementary file 8 — Supplementary Material 8 [file 12877_2023_4085_MOESM8_ESM.doc]

supplement Table 3. Meta-analysis of the risk factors of osteosarcopenia.

| Risk factors | Number of studies | Heterogeneity test | | Effect model | Results of meta-analysis | | *Z*-value | *P*-value |
| --- | --- | --- | --- | --- | --- | --- | --- | --- |
|  |  | *I*^2^/% | *P*-value |  | Pooled effect size | 95% *CI* |  |  |
| Female | 3 | 82 | 0.004 | random | 5.10 | 2.37-10.98 | 4.16 | <0.0001 |
| Age | 4 | 87 | <0.0001 | random | 1.12 | 1.03-1.21 | 2.65 | 0.008 |
| Fracture | 3 | 0 | 0.64 | fixed | 2.92 | 1.62-5.25 | 3.58 | 0.0003 |
| PHT | 3 | 92 | <0.00001 | random | 2.41 | 0.59-9.87 | 1.22 | 0.22 |
| BMI | 3 | 97 | <0.00001 | random | 1.01 | 0.63-1.62 | 0.04 | 0.97 |

PTH: parathyroid hormone; BMI: body mass index
