# Supplementary material for: Prevalence and risk factors of osteosarcopenia: a systematic review and meta-analysis
Source: BMC Geriatr. 2023 Jun 15;23:369. doi: 10.1186/s12877-023-04085-9 (PMC10273636; doi:10.1186/s12877-023-04085-9)
Supplement: Supplementary file 9 — Supplementary Material 9 [file 12877_2023_4085_MOESM9_ESM.doc]

supplement Table 4. Search terms and strategy.

This appendix provides search dates, search strings for the databases (PubMed, Embase, Cochrane Library, and Web of Science).

1. **PubMed**

| #No. | Search Details | Results |
| --- | --- | --- |
| #1 | "Osteoporosis"[MeSH Terms] | 60,215 |
| #2 | "Osteoporosis"[MeSH Terms] OR "Osteoporosis"[All Fields] OR "Osteoporoses"[All Fields] OR "osteoporosis, postmenopausal"[MeSH Terms] OR ("Osteoporosis"[All Fields] AND "postmenopausal"[All Fields]) OR "postmenopausal osteoporosis"[All Fields] OR "osteoporosis post traumatic"[Title/Abstract] OR "osteoporosis post traumatic"[Title/Abstract] OR ("Post-Traumatic"[All Fields] AND "Osteoporoses"[Title/Abstract]) OR "post traumatic osteoporosis"[Title/Abstract] OR "osteoporosis senile"[Title/Abstract] OR (("Osteoporosis"[MeSH Terms] OR "Osteoporosis"[All Fields] OR "Osteoporoses"[All Fields] OR "osteoporosis, postmenopausal"[MeSH Terms] OR ("Osteoporosis"[All Fields] AND "postmenopausal"[All Fields]) OR "postmenopausal osteoporosis"[All Fields]) AND "Senile"[Title/Abstract]) OR (("Senile"[All Fields] OR "seniles"[All Fields] OR "senility"[All Fields]) AND "Osteoporoses"[Title/Abstract]) OR (("Osteoporosis"[MeSH Terms] OR "Osteoporosis"[All Fields] OR "Osteoporoses"[All Fields] OR "osteoporosis, postmenopausal"[MeSH Terms] OR ("Osteoporosis"[All Fields] AND "postmenopausal"[All Fields]) OR "postmenopausal osteoporosis"[All Fields]) AND "Involutional"[Title/Abstract]) OR "senile osteoporosis"[Title/Abstract] OR "osteoporosis age related"[Title/Abstract] OR "osteoporosis age related"[Title/Abstract] OR "bone loss age related"[Title/Abstract] OR "age related bone loss"[Title/Abstract] OR "age related bone losses"[Title/Abstract] OR "bone loss age related"[Title/Abstract] OR ((("bone and bones"[MeSH Terms] OR ("Bone"[All Fields] AND "bones"[All Fields]) OR "bone and bones"[All Fields] OR "Bone"[All Fields]) AND "Losses"[All Fields]) AND "Age-Related"[Title/Abstract]) OR "age related osteoporosis"[Title/Abstract] OR "age related osteoporosis"[Title/Abstract] OR "age related osteoporoses"[Title/Abstract] OR (("Osteoporosis"[MeSH Terms] OR "Osteoporosis"[All Fields] OR "Osteoporoses"[All Fields] OR "osteoporosis, postmenopausal"[MeSH Terms] OR ("Osteoporosis"[All Fields] AND "postmenopausal"[All Fields]) OR "postmenopausal osteoporosis"[All Fields]) AND "Age-Related"[Title/Abstract]) | 98,482 |
| #3 | #1 or #2 | 98,482 |
| #4 | "Sarcopenia"[MeSH Terms] | 7,376 |
| #5 | "Sarcopenias"[Title/Abstract] | 2 |
| #6 | #4 or #5 | 7,376 |
| #7 | #3 and #6 | 501 |
| #8 | "Risk Factors"[MeSH Terms] | 923,151 |
| #9 | "factor risk"[Title/Abstract] OR "risk factor"[Title/Abstract] OR "social risk factors"[Title/Abstract] OR (("Factor"[All Fields] OR "factor s"[All Fields] OR "Factors"[All Fields]) AND "social risk"[Title/Abstract]) OR "factors social risk"[Title/Abstract] OR "risk factor social"[Title/Abstract] OR "risk factors social"[Title/Abstract] OR "social risk factor"[Title/Abstract] OR "health correlates"[Title/Abstract] OR "correlates health"[Title/Abstract] OR "population at risk"[Title/Abstract] OR "populations at risk"[Title/Abstract] OR "risk scores"[Title/Abstract] OR "risk score"[Title/Abstract] OR "score risk"[Title/Abstract] OR "risk factor scores"[Title/Abstract] OR "risk factor score"[Title/Abstract] OR "score risk factor"[Title/Abstract] | 279,286 |
| #10 | #8 or #9 | 1,080,818 |
| #11 | "Prevalence"[MeSH Terms] | 329,110 |
| #12 | "Prevalences"[Title/Abstract] OR "period prevalence"[Title/Abstract] OR "period prevalences"[Title/Abstract] OR "prevalence period"[Title/Abstract] OR "point prevalence"[Title/Abstract] OR "point prevalences"[Title/Abstract] OR "prevalence point"[Title/Abstract] | 22,706 |
| #13 | #11 or #12 | 341,238 |
| #14 | #10 or #13 | 1,321,786 |
| #15 | #7 and #14 | 164 |

1. **Embase**

| #No. | Query | Results |
| --- | --- | --- |
| #1 | 'Osteoporosis'/exp | 144,749 |
| #2 | 'Osteoporoses':ti,ab OR 'Osteoporosis, Post-Traumatic ':ti,ab OR 'Osteoporosis, Post Traumatic':ti,ab OR 'Post-Traumatic Osteoporoses':ti,ab OR 'Post-Traumatic Osteoporosis':ti,ab OR 'Osteoporosis, Senile':ti,ab OR 'Osteoporoses, Senile':ti,ab OR 'Senile Osteoporoses':ti,ab OR 'Osteoporosis, Involutional':ti,ab OR 'Senile Osteoporosis':ti,ab OR 'Osteoporosis, Age-Related':ti,ab OR 'Osteoporosis, Age Related':ti,ab OR 'Bone Loss, Age-Related':ti,ab OR 'Age-Related Bone Loss':ti,ab OR 'Age-Related Bone Losses':ti,ab OR 'Bone Loss, Age Related':ti,ab OR 'Bone Losses, Age-Related':ti,ab OR 'Age-Related Osteoporosis':ti,ab OR 'Age Related Osteoporosis':ti,ab OR 'Age-Related Osteoporoses':ti,ab OR 'Osteoporoses, Age-Related':ti,ab | 2,195 |
| #3 | #1 OR #2 | 145,387 |
| #4 | 'sarcopenia'/exp | 15,775 |
| #5 | 'sarcopenias':ti,ab | 27 |
| #6 | #4 OR #5 | 15,779 |
| #7 | #3 AND #6 | 1,391 |
| #8 | 'risk factor'/exp | 1,207,430 |
| #9 | 'factor, risk':ab,ti OR 'risk factors':ab,ti OR 'social risk factors':ab,ti OR 'factor, social risk':ab,ti OR 'factors, social risk':ab,ti OR 'risk factor, social':ab,ti OR 'risk factors, social':ab,ti OR 'social risk factor':ab,ti OR 'health correlates':ab,ti OR 'correlates, health':ab,ti OR 'population at risk':ab,ti OR 'populations at risk':ab,ti OR 'risk scores':ab,ti OR 'risk score':ab,ti OR 'score, risk':ab,ti OR 'risk factor scores':ab,ti OR 'risk factor score':ab,ti OR 'score, risk factor':ab,ti | 803,176 |
| #10 | #8 OR #9 | 1,492,222 |
| #11 | 'prevalence'/exp | 880,096 |
| #12 | 'prevalences':ab,ti OR 'period prevalence':ab,ti OR 'period prevalences':ab,ti OR 'prevalence, period':ab,ti OR 'point prevalence':ab,ti OR 'point prevalences':ab,ti OR 'prevalence, point':ab,ti | 28,656 |
| #13 | #11 OR #12 | 889,022 |
| #14 | #10 OR #13 | 2,183,100 |
| #15 | #7 AND #14 | 577 |

1. **Cochrane Library**

| #No. | Search | Hits |
| --- | --- | --- |
| #1 | MeSH descriptor: [Osteoporosis] explode all trees |  |
| #2 | (Osteoporoses):ti,ab,kw OR (Osteoporosis, Post-Traumatic):ti,ab,kw OR (Osteoporosis, Post Traumatic):ti,ab,kw OR (Post-Traumatic Osteoporoses):ti,ab,kw OR (Post-Traumatic Osteoporosis):ti,ab,kw |  |
| #3 | (Osteoporosis, Senile):ti,ab,kw OR (Osteoporoses, Senile):ti,ab,kw OR (Senile Osteoporoses):ti,ab,kw OR (Osteoporosis, Involutional):ti,ab,kw OR (Senile Osteoporosis):ti,ab,kw |  |
| #4 | (Osteoporosis, Age-Related):ti,ab,kw OR (Osteoporosis, Age Related):ti,ab,kw OR (Bone Loss, Age-Related):ti,ab,kw OR (Age-Related Bone Loss):ti,ab,kw OR (Age-Related Bone Losses):ti,ab,kw |  |
| #5 | (Bone Loss, Age Related):ti,ab,kw OR (Bone Losses, Age-Related):ti,ab,kw OR (Age-Related Osteoporosis):ti,ab,kw OR (Age Related Osteoporosis):ti,ab,kw OR (Age-Related Osteoporoses):ti,ab,kw |  |
| #6 | (Osteoporoses, Age-Related):ti,ab,kw |  |
| #7 | #1 OR #2 OR #3 OR #4 OR #5 OR #6 |  |
| #8 | MeSH descriptor: [Sarcopenia] explode all trees |  |
| #9 | (Sarcopenias):ti,ab,kw |  |
| #10 | #8 OR #9 |  |
| #11 | MeSH descriptor: [Risk Factors] explode all trees |  |
| #12 | (Factor, Risk):ti,ab,kw OR (Risk Factor):ti,ab,kw OR (Social Risk Factors):ti,ab,kw OR (Factor, Social Risk):ti,ab,kw OR (Factors, Social Risk):ti,ab,kw |  |
| #13 | (Risk Factor, Social):ti,ab,kw OR (Risk Factors, Social):ti,ab,kw OR (Social Risk Factor):ti,ab,kw OR (Health Correlates):ti,ab,kw OR (Correlates, Health):ti,ab,kw |  |
| #14 | (Population at Risk):ti,ab,kw OR (Populations at Risk):ti,ab,kw OR (Risk Scores):ti,ab,kw OR (Risk Score):ti,ab,kw OR (Score, Risk):ti,ab,kw |  |
| #15 | (Risk Factor Scores):ti,ab,kw OR (Risk Factor Score):ti,ab,kw OR (Score, Risk Factor):ti,ab,kw |  |
| #16 | #11 OR #12 OR #13 OR #14 OR #15 |  |
| #17 | MeSH descriptor: [Prevalence] explode all trees |  |
| #18 | (Prevalences):ti,ab,kw OR (Period Prevalence):ti,ab,kw OR (Period Prevalences):ti,ab,kw OR (Prevalence, Period):ti,ab,kw OR (Point Prevalence):ti,ab,kw OR (Point Prevalences):ti,ab,kw |  |
| #19 | (Prevalence, Point):ti,ab,kw |  |
| #20 | #17 OR #18 OR #19 |  |
| #21 | #16 OR #20 |  |
| #22 | #7 AND #10 AND #21 | 13 |

1. **Web of Science**

| #No. | Search strategy | Numbers |
| --- | --- | --- |
| #1 | TS=（Osteoporosis OR Osteoporoses OR Osteoporosis,Post-Traumatic OR Osteoporosis, Post Traumatic OR Post-Traumatic Osteoporoses OR Post-Traumatic Osteoporosis OR Osteoporosis, senile OR Osteoporoses, senile OR senile Osteoporoses OR Osteoporosis, Involutional OR Senile Osteoporosis OR Osteoporosis, Age-Related OR Osteoporosis, Age Related OR Bone Loss, Age-Related OR Age-Related Bone Loss OR Age-Related Bone Loss* OR Bone Loss, Age Related OR Bone Loss*, Age-Related OR Age-Related Osteoporosis OR Age Related Osteoporosis OR Age-Related Osteoporoses OR Osteoporoses, Age-Related） | 182,219 |
| #2 | TS=( Sarcopenia OR Sarcopenia*) | 22,395 |
| #3 | TS=(Risk factor* OR Factor, Risk OR Risk Factor OR Social Risk Factor* OR Factor, Social Risk OR Factor*, Social Risk OR Risk Factor, Social OR Risk Factor*, Social OR Social Risk Factor OR Health Correlates OR Correlates, Health OR Population at Risk OR Population* at Risk OR Risk Score* OR Risk Score OR Score, Risk OR Risk Factor Score* OR Risk Factor Score OR Score, Risk Factor) | 2,997,031 |
| #4 | TS=( Prevalence OR Prevalence* OR Period Prevalence OR Period Prevalence* OR Prevalence, Period OR Point Prevalence OR Point Prevalence* OR Prevalence, Point) | 1,448,946 |
| #5 | #4 OR #3 | 3,950,835 |
| #6 | #5 AND #2 AND #1 | 1,155 |

Rearch date：April 24^th^, 2022
